# High-Density Lipoprotein (HDL) in Allergy and Skin Diseases: Focus on Immunomodulating Functions

**DOI:** 10.3390/biomedicines8120558

**Published:** 2020-12-01

**Authors:** Athina Trakaki, Gunther Marsche

**Affiliations:** 1 Otto Loewi Research Center for Vascular Biology, Immunology and Inflammation, Division of Pharmacology, Medical University of Graz, Universitätsplatz 4, 8010 Graz, Austria; 2 BioTechMed Graz, Mozartgasse 12/II, 8010 Graz, Austria

**Keywords:** high-density lipoprotein, HDL composition, HDL function, allergy, skin disease, psoriasis, allergic rhinitis, atopic dermatitis, allergic asthma, immunomodulation

## Abstract

From an evolutionary perspective, lipoproteins are not only lipid transporters, but they also have important functions in many aspects of immunity. High-density lipoprotein (HDL) particles are the most abundant lipoproteins and the most heterogeneous in terms of their composition, structure, and biological functions. Despite strong evidence that HDL potently influences the activity of several immune cells, the role of HDL in allergies and skin diseases is poorly understood. Alterations in HDL-cholesterol levels have been observed in allergic asthma, allergic rhinitis, atopic dermatitis (eczema), psoriasis, urticaria, and angioedema. HDL-associated apolipoprotein (apo) A-I, apoA-IV, and apoC-III, and lyso-phosphatidylcholines potently suppress immune cell effector responses. Interestingly, recent studies provided evidence that allergies and skin diseases significantly affect HDL composition, metabolism, and function, which, in turn, could have a significant impact on disease progression, but may also affect the risk of cardiovascular disease and infections. Interestingly, not only a loss in function, but also, sometimes, a gain in function of certain HDL properties is observed. The objective of this review article is to summarize the newly identified changes in the metabolism, composition, and function of HDL in allergies and skin diseases. We aim to highlight the possible pathophysiological consequences with a focus on HDL-mediated immunomodulatory activities.

## 1. Introduction

The prevalence of allergic and inflammatory skin diseases has dramatically increased in recent decades, a fact that is linked to changes in environmental exposures and lifestyle practices [1,2]. Despite strong evidence that high-density lipoprotein (HDL) potently influences the activity of several immune cells, including monocytes, macrophages, eosinophils, and neutrophils [3,4], the role of HDL particles in allergies and skin diseases is still poorly understood [5,6]. HDL particles are regarded as cholesterol transporters, mainly mediating the reverse cholesterol transport from extrahepatic peripheral tissues back to the liver. Although their association with reduced cardiovascular risk is well established [7,8,9,10], HDL-cholesterol raising therapies failed to improve the cardiovascular outcome [11,12,13], and recent studies challenged the causal role of low HDL-cholesterol levels in cardiovascular diseases [14].

HDL is quantitatively the most important lipoprotein in most species and mechanistic evidence points towards a role of HDL in physiological immune function [15], while low HDL-cholesterol levels are associated with a high risk of autoimmune disease in individuals from the general population [16]. In this context, the potential role of HDL in other diseases, such as infections and allergies, but also skin diseases, has gained much attention.

Apolipoprotein (apo) A-I is the main structural and functional apoprotein of HDL [17], and it plays a key role in the induction of cholesterol efflux from cells [18]. The interaction of HDL with cells results in cholesterol depletion in specific membrane microdomains enriched in cholesterol and sphingolipids, named lipid rafts, a mechanism that is known to disrupt raft-dependent signaling [19,20]. Their main role is the compartmentalization of molecules to form functional platforms for biological processes, such as toll-like receptors (TLRs) [21]. The lipid composition of rafts determines their function; the modification of lipid raft composition can modulate raft-dependent signaling due to protein delocalization and alter immune cell biological functions [21]. HDL, along with apoA-I, have been shown to disrupt the plasma membrane of lipid rafts in antigen presenting cells, leading to the inhibition of their capacity to stimulate T cell activation [22]. On the other hand, lyso-phosphatidylcholine, which is one of the main phospholipid subtypes carried by HDL particles [23], has been shown to directly activate TLRs 1, 2, and 4 in the absence of classical TLR-ligands; however, in the presence of classical TLR-ligands, it induces an anti-inflammatory phenotype [24]. TLRs are expressed by a plethora of cells in the skin, including Langerhans cells, keratinocytes, and several immune cells [25,26]. Furthermore, TLRs are implicated in the pathogenesis of atopic dermatitis [25,27,28] and psoriasis [25,28].

The composition and particle distribution of HDL are significantly altered in allergic and skin diseases, which ultimately lead to altered HDL functionality and an altered ability of HDL to modulate immune cell effector responses [4,29,30,31,32,33,34,35,36]. Here, we will provide an updated review on the novel identified activities of HDL in allergic diseases, including allergic asthma and allergic rhinitis, and common skin diseases. We will focus on how HDL modulates the immune cell function of certain cell types that are implicated in the pathogenesis of allergic and skin diseases.

## 2. HDL Metabolism, Composition and Function

**Figure 1:**
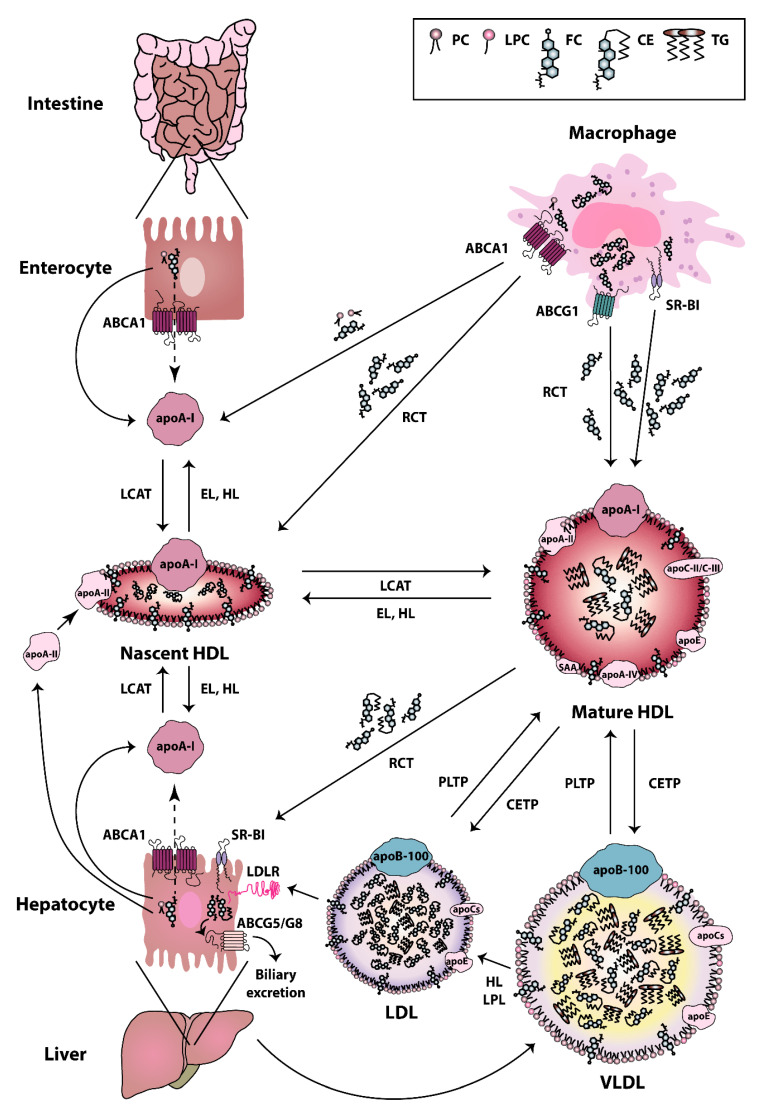
High-density lipoprotein (HDL) metabolism. HDL metabolism is a multistep process involving (i) the secretion of lipid-free apolipoproteins by the liver or intestine, (ii) the acquisition of cholesterol and phospholipids via ATP-binding cassette (ABC) transporter A1 (ABCA1), ABCG1, and scavenger receptor class B type I (SR-BI), (iii) the maturation by lecithin cholesterol acyltransferase (LCAT)-mediated cholesterol esterification and (iv) the final uptake of lipids by the liver. Cholesterol uptake is either mediated directly via SR-BI, or indirectly via cholesteryl-ester transfer protein (CETP)-mediated transfer of cholesteryl ester to very low-density lipoproteins (VLDL) and low-density lipoproteins (LDL) and uptake by the LDL-Receptor. The liver excretes then cholesterol into the bile, either directly via the action of ABCG5/G8 transporters, or indirectly following oxidation to bile acid and secretion via ABCB11 [69,70]. Abbreviations represent: ABCA1, ATP-binding cassette subfamily A member 1; ABCG1, ATP-binding cassette subfamily G member 1; ABCG5, ATP-binding cassette subfamily G member 5; ABCG8, ATP-binding cassette subfamily G member 8; apoA-I, apolipoprotein A-I; apoA-II, apolipoprotein A-II; apoA-IV, apolipoprotein A-IV; apoB-100, apolipoprotein B-100; apoC, apolipoprotein C; apoC-II, apolipoprotein C-II; apoC-III, apolipoprotein C-III; apoE, apolipoprotein E; CE, cholesteryl ester; CETP, cholesteryl ester transfer protein; EL, endothelial lipase; FC, free cholesterol; HDL, high-density lipoprotein; HL, hepatic lipase; LCAT, lecithin-cholesterol acyltransferase; LDL, low-density lipoprotein; LDLR, low-density lipoprotein receptor; LPC, lyso-phosphatidylcholine; LPL, lipoprotein lipase; PC, phosphatidylcholine; PLTP, phospholipid transfer protein; RCT, reverse cholesterol transport; SAA, serum amyloid A; SR-BI, scavenger receptor class B type I; TG, triglyceride; VLDL, very low-density lipoprotein.

HDL particles are heterogeneous in terms of their composition, structure, and biological functions. The biogenesis of HDL is a complex process [37]. The first step in HDL formation is the secretion of apoA-I by the liver and intestine [38]. Secreted apoA-I interacts thereafter with ATP-binding cassette (ABC) transporter A1 (ABCA1), which leads to the rapid recruitment of cellular phospholipids and cholesterol to lipid-poor apoA-I. Afterwards, the lipidated apoA-I is gradually converted into discoidal HDL particles, containing unesterified cholesterol [39]. The acquisition of cholesterol and the esterification of free cholesterol to cholesteryl esters by the enzyme lecithin cholesterol acyltransferase (LCAT) [40] lead to the evolution of more mature, large-sized particles [41]. HDLs are extensively remodeled in the bloodstream via the action of lipid transfer proteins, such as cholesteryl-ester transfer protein (CETP), LCAT, and phospholipid transfer protein (PLTP). CETP is responsible for the bidirectional transfer of cholesteryl esters and triglycerides between plasma lipoproteins [42]. PLTP mediates the phospholipid transfer among lipoproteins [43], which converts HDL into larger and smaller particles [44,45]. In addition, certain lipases, such as endothelial and hepatic lipases, as well as lipid exchange with cellular transporters, such as ABCA1 and ABCG1, and scavenger receptor class B type I (SR-BI), affect HDL maturation and catabolism [46,47]. Plasma endothelial and hepatic lipases have specificity for phospholipids and triglycerides of large HDL and apoB-containing lipoproteins remnants [48,49]. The hydrolysis of triglycerides and phospholipids of HDL leads to the conversion of HDL2 into HDL3 and pre-beta HDL [45]. ABCA1 and ABCG1 both play a crucial role in the reverse cholesterol transport pathway. ABCA1 is responsible for the transfer of cellular phospholipids and cholesterol to lipid poor apoA-I, while ABCG1 promotes cholesterol efflux to more mature HDL particles [45]. SR-BI is primarily expressed by the liver, but it is also found in other tissues [50]. SR-BI absorbs cholesterol and cholesteryl ester of HDL without causing HDL degradation in the liver [51]. SR-BI also promotes cholesterol efflux from macrophages and other cell types to HDL particles, thus acting as a bidirectional cholesterol transporter [52] (Figure 1). HDL can be divided into the relatively cholesterol-rich, larger, spherical, and less dense HDL2 particles (1.063–1.125 g/mL), and the more protein-rich, smaller, and denser HDL3 particles (1.125–1.21 g/mL) [53]. The latter particles appear to display the most potent atheroprotective properties [54]. In addition to apoA-I and apoA-II, which are the main protein components, HDL particles contain other less abundant proteins, including apoA-IV, apoC-II, apoC-III, apoE, and serum amyloid A (SAA) [55]. Some studies reported that more than 100 different proteins are associated with HDL, which suggests a multiple functionality for the HDL particles [56]. Not all protein species are present on every single HDL particle, and most proteins are only carried by a small fraction of the HDL particles [57]. However, there is recent evidence that the HDL proteome of mature HDL3 and HDL2 subclasses may be less complex than expected and contains less than 20 proteins after extensive purification [58]. This seems to contradict other publications that assume a much more complex HDL proteome [57,59,60]. However, in these publications, not only HDL2 and HDL3 were isolated and investigated, but also pre-beta HDL. Therefore, the different number of identified proteins is due to the other purification strategies of the HDL subclasses. Moreover, more than 200 lipid species have been identified in HDL particles [61,62], including cholesterol (free or esterified), triglycerides, phospholipids, lyso-phospholipids, and sphingolipids [54]. The structure and dynamic properties of lipids significantly depend on their location in the particle (surface, intermediate region, core). Not only hydrophobicity, but also conformational entropy of the molecules, are the driving forces in the formation of the HDL structure [63]. For example, apoA-I has a strong preference for binding to HDL (d = 8–12 nm), as compared to larger, less curved low-density lipoproteins (LDL) (d = 20–24 nm) or very low-density lipoproteins (VLDL) (d = 40–100 nm). The high radius of curvature of HDL as compared to other lipoproteins causes packing defects of phospholipids, and this is the reason why other lipids and amphipathic proteins associate with HDL when compared to other lipoproteins [64]. In addition to the promotion of cellular cholesterol efflux, HDL particles display a number of anti-inflammatory activities, such as cytoprotective, vasodilatory, anti-oxidative, anti-thrombotic, and anti-infectious activities [54]. Among the HDL associated enzymes, paraoxonase (PON) is known to exert a protective effect against oxidative damage of circulating cells and lipoproteins and to modulate the susceptibility of HDL to atherogenic modifications, such as homocysteinylation and glycation, even exerting an anti- inflammatory role [65]. Other HDL-associated enzymes are LCAT, platelet-activating factor-acetyl hydrolase (PAF-AH) (also known as lipoprotein-associated phospholipase A2 (Lp-PLA2)), and PLTP. Among these, LCAT is responsible for the esterification of free cholesterol to cholesteryl esters [40]. PAF-AH is mainly associated with low-density lipoproteins, however about 30% is also associated with HDL [66]. PAF-AH is the major enzyme catabolizing platelet-activating factor (PAF) and PAF-like lipids, which are potent inflammatory mediators [67,68]. PLTP is a lipid transfer protein that is involved in the remodeling of HDL particles [44] and it has been reported to contribute to the anti-oxidative HDL activity [57].

## 3. Potential Role of HDL in Atopic Allergic Diseases

### 3.1. Relation of HDL with Asthma

Allergic (or atopic) asthma is a chronic airway inflammatory disease and it is the most common form of asthma [71]. It is estimated that more than 330 million individuals worldwide suffer from asthma, with this number potentially increasing by a hundred million more by 2025 [72]. Asthma most commonly onsets in childhood, where it is often associated with seasonal allergic rhinitis and atopic dermatitis; however, one in four individuals may experience recurring or persisting symptoms in adulthood [73]. It is a complex disease, implicating multiple genetic and environmental factors, in which allergen exposure often induces intermittent attacks of coughing, wheezing, breathlessness, and airway hyper-reactivity [73]. Allergic asthma is associated with T helper cell type 2 immune responses, which promote eosinophilic inflammation and immunoglobulin E (IgE) production, through specific cytokine release [74].

Several studies so far have examined the relation of asthma with serum dyslipidemia, reporting positive [75,76,77,78], negative [79,80,81,82], or no association [83,84,85,86]. Accumulating evidence suggests an association of HDL-cholesterol with pulmonary function and pulmonary disorders [5]. Reports on serum HDL-cholesterol levels among asthmatic individuals vary, with some studies demonstrating higher [79,81,87], lower [88,89,90,91], or unchanged [76,80,92,93] levels in comparison to controls. A direct correlation between HDL-cholesterol levels and asthma could not yet be shown due to these contradictory data. In 2012 Yiallouros et al. reported that low levels of serum HDL-cholesterol in childhood are associated with an increased risk for asthma in adolescence, which suggests a potential role of HDL in the pathogenesis of pediatric asthma [89]. This is in line with a study by conducted by Cirillo et al., reporting that higher systemic levels of HDL are associated with less severe airflow obstruction in both asthmatics and healthy subjects [94]. These data are in agreement with a recent meta-analysis reporting significantly lower serum HDL-cholesterol in asthmatic in comparison to non-asthmatic children, although, in adults, the aforementioned difference was not significant [95]. Moreover, Barochia et al. reported that the concentration of large HDL particles in serum was positively correlated with the forced expiratory volume (FEV_1_) and lung function in atopic asthmatic subjects [96,97]. A few years later, the same group reported that the concentration of HDL particles was negatively correlated with the blood eosinophil number in atopic asthmatics, supporting the concept that serum HDL levels are linked to systemic type 2 inflammation in atopic asthma [98]. In addition to this, Rastogi et al. reported an inverse association between HDL-cholesterol and circulating monocytes, a cell population rapidly recruited to the lung upon inflammation in obese, adolescent asthmatics [99]. Moreover, Ouyang et al. demonstrated a sex association of allergic sensitization and HDL. In particular, the risk of allergic sensitization was four times higher in men when the HDL-cholesterol levels were below 40 mg/dL, while, in women, a less strong association was observed [100]. It has to be noted that other studies reported no association between asthma severity and HDL-cholesterol levels [101,102] and opposite findings were even reported in one study [103]. In this study, higher HDL-cholesterol levels were associated with decreased FEV_1_ and forced vital capacity (FVC) among healthy, male adolescents, which was not observed in female adolescents [103].

Besides HDL-cholesterol levels, Cirillo et al. reported a positive correlation of serum apoA-I levels with less severe airflow obstruction in asthmatic individuals [94]. Barochia et al. confirmed this, reporting that serum apoA-I was positively correlated with FEV_1_ and lung function in atopic asthmatics, which implied that circulating concentrations of apoA-I may have a protective effect on airflow obstruction in asthma [96]. The ApoA-I levels were decreased in bronchoalveolar lavage fluid of patients with moderate to severe asthma, which suggested an augmentation of disease severity due to lower apoA-I levels in the lung [104]. ApoA-I attenuates lipopolysaccharide (LPS)-mediated neutrophilic inflammation [105], which has been associated with an increased risk for asthma and wheezing [106,107]. In addition, its immunomodulatory and anti-inflammatory properties may be relevant for adaptive immune responses in allergic asthma, since apoA-I can suppress adaptive immunity through the inhibition of function, differentiation, and maturation of dendritic cells [22,108,109]. The ApoA-I levels are dramatically decreased in patients with chronic obstructive pulmonary disease and correlate with FEV_1_/FVC, which suggested a relationship to disease severity [110].

In addition to altered HDL-cholesterol and apoA-I levels, lower plasma paraoxonase and arylesterase activities have been observed in asthmatic patients [90,93]. Lp-PLA2 activity was negatively correlated with asthma severity score and it was lower in asthmatic women [111]. However, it has to be noted that only 20–30% of this enzyme is associated with HDL [66]. PAF, which is the substrate of Lp-PLA2, was detected in higher concentrations in sputum [112,113] and bronchoalveolar lavage fluid [113] of asthmatic subjects in comparison to non-asthmatic. Moreover, increased PAF levels were observed in the human plasma during acute asthma attacks [114,115,116] and upon allergen challenge [117], while the lung tissues of asthmatic patients have increased mRNA levels of PAF receptor [118].

In summary, due to contradictory data, an association between HDL-cholesterol levels and asthma could not yet be shown. However, most studies reported that a decrease in apoA-I levels is associated with an increased risk of asthma development, while higher apoA-I levels are associated with less severe airflow obstruction in asthmatics.

Interestingly, mice with a genetic deletion of apoA-I display a phenotype of increased lung inflammation and oxidative stress, along with enhanced airway hyperresponsiveness [119]. Studies using murine models of experimental asthma have identified a role for the apoA-I/ABCA1 pathway in mediating neutrophilic airway inflammation [120]; it was demonstrated that endogenous apoA-I negatively regulates ovalbumin-induced neutrophilic airway inflammation [121]. Furthermore, apoA-I mimetic peptides have been extensively studied in murine models of experimental asthma (reviewed in detail [120,122]). ApoA-I mimetic peptides attenuate the development of airway inflammation, remodeling, and hyperresponsiveness [105], and decrease eosinophil counts [123].

### 3.2. Allergic Rhinitis is Associated with Complex Alterations in HDL Composition and Function

Allergic rhinitis is an IgE-mediated disease, which is strongly linked to asthma and conjunctivitis [124,125]. Allergic rhinitis affects approximately 20% of the population and it is characterized by nasal itching, sneezing, watery discharge, and congestion [124,125]. Although originally considered as a disorder mainly localized to the nose and nasal passages, current evidence proposes that it may represent a component of a systemic airway disease of the entire respiratory tract [125]. Allergic rhinitis is caused by inhaled allergens in genetically predisposed individuals, mainly proteins and glycoproteins that are found in airborne particles, such as grass pollinosis, ragweed, and dust mite [124].

HDL-cholesterol is increased in allergic rhinitis children [79], and there is strong evidence that HDL plays an important role in allergic rhinitis. A study conducted by Vinding et al. reported that high HDL-cholesterol levels in children with asthma or allergic rhinitis were associated with a lower risk of sensitization against aeroallergens [126]. HDL particles are diverse in composition and structure, representing the basis for their functional heterogeneity [54]. Evaluation of allergic rhinitis-derived HDL particle distribution via gradient gel electrophoresis revealed a significant decrease in the HDL3 subclass in comparison to controls, while other HDL subclasses were not altered [29]. The change in HDL3 subclass was associated with an impaired ability of allergic rhinitis HDL to suppress monocyte cytokine secretion [29], which is consistent with reports of the HDL3 subclass being superior to the HDL2 subclass in inflammatory responses’ suppression [54]. Moreover, correlations of specific IgE levels of grass pollen were detected with the HDL2/HDL3 ratio and the small HDL3 subclass in allergic rhinitis patients [29].

Interestingly, recent findings suggested that allergic rhinitis has profound effects on the composition of HDL. HDL that is isolated from allergic rhinitis patients has a significantly reduced content of apoA-I and phosphatidylcholine, but an increased content of apoA-II, lyso-phosphatidylcholine, and triglycerides, in comparison to HDL isolated from non-allergic healthy controls [29]. The compositional alterations of HDL are closely linked to alterations of HDL functional properties [29]. Other studies reported an increase in apoA-I and apoA-II, which are the major HDL apolipoproteins, in the mucus proteome of allergic rhinitis patients, suggesting a direct modulation of the immune response by HDL apolipoproteins [127,128,129]. Increased apoA-I levels were also detected in nasal lavage fluids of subjects with persulfate-associated rhinitis after challenge with potassium persulfate [130]. Moreover, in allergic rhinitis patients, correlations of specific IgE levels of grass pollen were observed with apoE and SAA content of HDL [29]. It has been reported that specific IgE levels of house dust mite are associated with reduced odds for myocardial infarction [131] and reduced HDL-cholesterol [132]. In another study, allergic rhinitis was associated with metabolic syndrome [133]. A recent study conducted by Roula et al. reported decreased levels of the HDL apolipoprotein apoA-IV in allergic rhinitis patients in comparison to the controls. Importantly, apoA-IV is an endogenous anti-inflammatory protein that potently suppresses effector functions of eosinophils [134]. Moreover, Makino et al. identified the serum levels of apoA-IV to be significantly increased in sublingual immunotherapy treated in comparison to placebo treated seasonal allergic rhinitis patients [135]. ApoA-IV was negatively correlated with the clinical symptom-medication and quality of life scores. The authors concluded that apoA-IV might be a potential target molecule for the treatment of seasonal allergic rhinitis [135]. Moreover, Chung et al. reported that apoE was up-regulated in nasal secretomes that were obtained from chronic rhinosinusitis patients [136], a condition that is frequently associated with rhinitis [137], demonstrating that it can potentially be a biomarker of nasal mucosal inflammation [136], while increased levels of apoA-I were found in sinonasal mucosa obtained from chronic rhinosinusitis patients with nasal polyps [138].

Structural and compositional alterations in HDL affect HDL function in individuals suffering from allergic rhinitis. HDL that was isolated from allergic rhinitis patients showed an impaired anti-oxidative and anti-inflammatory capability, as well as an impaired ability to suppress pro-inflammatory cytokine secretion [29].

Of particular interest, HDL that was derived from allergic rhinitis patients showed an improved ability to suppress eosinophil effector responses upon eotaxin-2/CCL24 stimulation. This gain of anti-allergic activity of isolated HDL was linked to altered apoA-II, apoC-III, lyso-phosphatidylcholine and phosphatidylcholine content of HDL [29]. Moreover, sera from allergic rhinitis patients showed decreased CETP and paraoxonase activities and increased Lp-PLA2 activity [29]. Decreased serum paraoxonase levels were also observed in children with allergic rhinitis [139].

In summary, there is strong evidence that HDL plays an important role in allergic rhinitis. The evaluation of allergic rhinitis-derived HDL particle distribution revealed a significant decrease in the HDL3 subclass and profound effects on the composition of HDL. Alterations in the HDL structure and composition are linked to decreased anti-oxidative and anti-inflammatory properties, but an improved ability of HDL to suppress eosinophil effector responses.

## 4. HDL in Inflammatory Skin Diseases

The skin is one of the largest immunologic organs, while it is often a target for allergic and immunologic responses [140]. Immune-mediated skin diseases, such as contact dermatitis, atopic dermatitis, psoriasis, urticaria, angioedema, and autoimmune blistering disorders are becoming all the more common nowadays, while most of them are chronic and inflammatory with both environmental and genetic factors contributing [140]. Many skin disorders are known to be associated with dyslipidemia, while some of the dermatological therapies are also known to predispose to lipid abnormalities [6].

### 4.1. Atopic Dermatitis is Associated with Complex Alterations in HDL Composition and Function

Atopic dermatitis (or eczema) is the most common atopic disease in young children and the most common skin disease in childhood [141]. Atopic dermatitis comprises a common chronic inflammatory skin disease with heterogeneous clinical phenotypes that are determined by both genetic and epigenetic dispositions [142]. In more than half of the patients the disease starts before the age of 6, while a less frequent onset is observed after the age of 20 [143]. Atopic dermatitis has different onset patterns and disease course is associated with distinct clinical features, food intolerance, risk of concomitant allergic diseases, and impact of psychic factors on symptoms [143]. In the last years, associations of atopic dermatitis with other inflammatory diseases have been reported, including systemic lupus erythematosus, rheumatoid arthritis, inflammatory bowel disease [144], and increased cardiovascular risk [145,146,147,148,149].

Although there is evidence that HDL is an important modulator of the immune response, few studies have investigated the role of HDL in human atopic dermatitis. A study conducted by Schäfer et al. reported increased HDL-cholesterol levels in patients in comparison to controls [76]; however, another study by Agón-Banzo et al. on a pediatric population, along with the study by Trieb et al., reported no difference [35,150].

A further study reported that apoA-I was highly expressed in the horny layer of the skin of atopic dermatitis patients in comparison to controls and it was associated with the severity of specific eruptions [151]. In a recent study by Trieb et al., the composition of HDL was evaluated in atopic dermatitis patients and control subjects [35]. Interestingly, the authors identified complex HDL compositional alterations. Specifically, the authors observed a significant enrichment of atopic dermatitis-HDL in apoA-II, the acute-phase protein SAA, and phosphatidylinositol, while a trend towards increased sphingomyelin content of atopic dermatitis-HDL was also observed [35]. Moreover, a significant reduction in atopic dermatitis-HDL content of apoC-III, apoE, cholesteryl ester, free cholesterol, lyso-phosphatidylcholine (especially 16:0 species), and phosphatidylethanolamine was observed when compared to the control subjects [35].

Eosinophils comprise a cell subset inducing tissue damage in the inflammatory infiltrate within the dermis of atopic dermatitis patients [152]. The effector responses of HDL isolated from patients suffering from atopic dermatitis and healthy controls were evaluated in a previous study while using freshly isolated human eosinophils [35]. Eosinophils were stimulated with eotaxin-2/CCL24 in the presence or absence of HDL (isolated from patients suffering from atopic dermatitis and healthy controls) and morphological changes (evaluated by the change in shape via flow cytometry) or chemotaxis was monitored. Of particular interest, the majority of HDL that was isolated from atopic dermatitis patients increased agonist induced eosinophil effector responses when compared to control-HDL. The authors demonstrated that the HDL-associated apoC-III and lyso-phosphatidylcholine species 16:0 and 18:0 effectively suppressed eosinophil shape change and migration [35]. Interestingly, the HDL content of apoC-III and lyso-phosphatidylcholine species 16:0 and 18:0 was much lower in HDL that was isolated from atopic dermatitis patients, and it was linked to an impaired ability of HDL to supress eosinophil effector responses. Moreover, by performing a detailed correlation analysis between function and composition of HDL isolated from atopic dermatitis patients, the authors demonstrated that the HDL-triglyceride content was negatively associated with the HDL activity towards agonist-induced eosinophil shape change and migration. In contrast, the HDL-associated SAA was associated with the ability of HDL to suppress agonist-induced eosinophil shape change [35]. In addition, the HDL-associated paraoxonase activity was decreased in atopic dermatitis-HDL; however, no change was observed in the capacity of atopic dermatitis-HDL to mobilize cholesterol from cells, when compared to the control-HDL [35].

In conclusion, there is increasing evidence that atopic dermatitis is associated with profound alterations in the HDL composition, linked to the formation of dysfunctional HDL. In contrast to the HDL that was isolated from allergic rhinits patients [29], the ability of HDL to suppress eosinophil effector responses is suppressed in atopic dermatitis, which suggests disease specific links between HDL composition, dysfunction, and disease severity.

### 4.2. HDL in Psoriasis

Epidemiological and clinical studies have shown a consistent association of psoriasis with systemic metabolic disorders, including an increased prevalence of diabetes, obesity, and cardiovascular disease [153]. Psoriasis is a common chronic inflammatory skin disease, which affects approximately 2–3% of the population in Western countries [154], and it is equally prevalent in both sexes [155]. Psoriasis is characterized by the appearance of red scaly plaques, affecting any part of the body, but predominately appearing over elbows and knees, on the scalp, the perianal, and the umbilical region [154]. The pathogenesis of psoriasis is complex, involving the activation of plasmacytoid dendritic cells by epidermal antigens due to skin trauma as the initial step [156], followed by maturation of myeloid dendritic cells, which promote the differentiation of T cells into Th1 and Th17 cells, via the secretion of interleukin (IL)-6, IL-12, and IL-23 [157]. Pro-inflammatory cytokines and chemokines that are produced by activated keratinocytes are able to recruit a variety of inflammatory cells from the circulation, leading to a “vicious cycle” of excessive immune response [158].

Already in the 90s, studies reported alterations in plasma lipids [159,160] and HDL-apolipoprotein content [159] in psoriatic children. The results from studies evaluating, among others, HDL-cholesterol levels between psoriasis patients and controls, vary greatly, reporting either increased [161,162,163,164], decreased [30,31,33,34,165,166,167,168,169,170,171,172,173,174,175,176,177,178,179,180,181,182,183,184,185,186,187,188] or unchanged [32,189,190,191,192,193,194,195,196,197,198,199,200,201,202,203,204,205] levels. Interestingly, Yu et al. demonstrated an increase in the small HDL subclass in psoriasis patients, which was associated with aortic inflammation [31], while Tom et al. reported a decrease in the large HDL subclass in paediatric psoriasis patients in comparison to controls, but no change in the small or medium HDL subclasses was observed [32].

Anti-inflammatory, anti-psoriatic therapies appear to induce complex changes in the HDL-cholesterol levels. The current treatment options include topicals, such as corticosteroids, as well as agents such as anthralin, synthetic vitamin D3 and vitamin A; phototherapy, including broad and narrowband-ultraviolet B (UVB), laser UVB, and psoralen and ultraviolet A (PUVA); systemics, such as methotrexate, cyclosporine, and retinoid receptor inhibitors (acitretin); and, biological therapeutics targeting tumor necrosis factor (TNF)-alpha, IL-23p40, or IL-17 [206]. Tofacitinib, an oral janus kinase (JAK) inhibitor [207,208,209], metformin, an anti-inflammatory agent activating adenosine monophosphate-activated protein kinase (AMPK) [210], and adalimumab [211], etanercept [212], or other TNF-alpha blockers [213] appear to increase HDL-cholesterol levels; whereas, topical [178] or systemic treatment with methotrexate [214] or acitretin [215] seem to decrease HDL-cholesterol levels. Etanercept [216], anti-IL17A antibodies, such as ixekizumab [217] and secukinumab [218], or other biologic treatments [219], appear not to affect HDL-cholesterol levels. In 2014, Holzer et al. demonstrated an increase in the large HDL subclass in psoriasis patients upon systemic and/or topical treatment in comparison to baseline [33]. In 2018 Mehta et al. reported an increase in the HDL-particle number at 12 weeks of phototherapy and a trend towards increase after adalimumab treatment, however at 52 weeks of adalimumab treatment a significant reduction of the HDL-particle number was observed in comparison to the baseline [220]; while, treatment with secukinumab induced no change in HDL particle number and size [218]. In 2017, Wolk et al. reported a striking increase in total HDL particles upon different dosages of tofacitinib for four or 16 weeks in comparison to baseline measurements; specifically the authors observed an increase in the small HDL subclass, while medium and large HDL subclasses remained unchanged [207]. Much like the effects of systemic or biological therapeutics on HDL-cholesterol levels, the distribution of HDL particles is also affected, since it appears to be dependent not only on the pharmacological agent, but also on the duration of treatment. In 2012, a study evaluated several aspects of HDL composition in HDL that was isolated by ultracentrifugation in a small cohort of psoriasis patients receiving mainly topical treatment [30]. Among the main HDL-associated proteins and lipids, the authors were able to demonstrate a reduction in the levels of apoA-I, total cholesterol, cholesteryl esters, free cholesterol, phosphatidylcholine and sphingomyelin, and an increase in the levels of apoA-II and acute-phase proteins, such as SAA and α-1-antitrypsin, in HDL that is derived from psoriasis patients in comparison to the controls [30]. However, previous studies reported increased [161,162], decreased [165,176,178], or unchanged [32,167,185,189,199,205] apoA-I levels in psoriasis patients compared to healthy controls.

Due to these contradictory data, no direct and clear correlation between psoriasis and HDL quantity, particle size distribution, or composition has been demonstrated so far. Further studies are necessary in order to understand the observed effects.

However, the effects of anti-psoriatic therapy on some metrics of HDL function are more evident. During the last decade, studies coming from several groups have demonstrated significantly impaired HDL-cholesterol efflux capacity in psoriasis patients in comparison to controls [30,32,33,34], which appeared to recover upon systemic and/or topical treatment [33]. HDL-mediated cholesterol efflux capacity was negatively associated with psoriasis area severity index score [30,33], being significantly impaired in patients with higher psoriasis area severity index score [32], while it was positively associated with impaired levels of apoA-I, phosphatidylcholine, sphingomyelin [30], and total phospholipid HDL content [33]. A recent study conducted by Mehta et al. indicated reduced cholesterol efflux capacity at 52 weeks of adalimumab treatment [220]. The JAK inhibitor tofacitinib showed no change in cholesterol efflux capacity upon a 16-week treatment, as was recently reported by Wolk et al. [207], while secukinumab treatment for 12 or 52 weeks also induced no change [218].

Furthermore, the anti-inflammatory potential of HDL was markedly impaired in psoriasis patients when compared to controls [185]. Of particular interest, a study identified apoA-I, HDL-cholesterol, and HDL-cholesterol efflux capacity to be predictors of noncalcified coronary burden in psoriasis [221]. Moreover, an improved HDL-associated Lp-PLA2 activity in patients in comparison to controls was observed [30,33], which was positively correlated with the psoriasis area severity index score [30]. Upon systemic and/or topical treatment or biologic treatment, patients showed improved LCAT activity in comparison to the baseline [33,207].

In conclusion, recent studies provided clear evidence that psoriasis affects HDL composition that is linked to a significantly impaired capability to mobilize cholesterol from macrophages, a crucial step in reverse cholesterol transport. HDL quantity and other functionalities assessed in psoriasis patients, including paraoxonase activity and anti-oxidative properties of HDL, are contradictory. Interestingly, as demonstrated by Asefi et al., PON 55 methionine allele is a risk factor for psoriasis [222]. However, in psoriasis patients, unchanged [30], improved [204,205], or impaired [33,185,186,203,223,224,225] paraoxonase activity was observed in comparison to healthy controls. Rocha-Pereira et al. showed a significantly reduced total anti-oxidant potential in patients in comparison to controls [183], while others observed no difference in the anti-oxidant HDL capacity [30,33].

All of these data only suggest a loss of cholesterol efflux capacity of HDL in patients with psoriasis, corresponding to the increased cardiovascular risk of these patients, while other metrics of HDL quantity and quality are inconclusive. This also suggests that studying the influence of anti-psoriatic agents on HDL-cholesterol efflux capacity may help to identify treatment strategies with beneficial effects on long-term cardiovascular outcome.

### 4.3. HDL in Urticaria

Urticaria is a common chronic clinical condition that presents with angioedema, wheals (hives), or both [226], occurring in 15–25% of individuals at some point of life [227], and it is one of the 10 most common dermatoses [228]. Urticaria presents a high burden for the patient due to its chronic course and the difficulties in diagnosis and treatment, ultimately reducing performance and quality of life [229]. Urticaria is characterized by a recurrent, pruritic, wheals of pale, central swelling, and surrounding epidermal erythema, with the potential of appearance over any part of the body and with lesions ranging in size from a few millimeters to several centimeters [227]. Mast cells are the primary effector cells in urticaria, and their degranulation leads to a rapid release of a plethora of inflammatory mediators, such as leukotrienes, prostaglandins, and histamine, which, in turn, cause vasodilation and leakage of plasma below and in the skin. A more delayed secretion of inflammatory cytokines follows, including IL-4, IL-5, and TNF-alpha, potentially leading to further inflammatory responses and longer lasting lesions [230]. The pathogenesis, classification, diagnosis, and treatment options of urticaria have been extensively reviewed elsewhere [226,227,231], and they are not in the focus of the current review.

A study conducted by Amin and Rushdy has recently demonstrated significantly decreased serum levels of HDL-cholesterol in chronic spontaneous urticaria patients in comparison to control subjects, which were negatively associated with TNF-alpha [232]. Similarly, another study also demonstrated a reduction of serum HDL-cholesterol levels in chronic spontaneous urticaria patients in comparison to the controls, with HDL-cholesterol levels being negatively associated with right and left carotid intima media thickness, discussing the likelihood of a potentially increased atherosclerosis risk in those patients [233]. Further studies are warranted in order to confirm a potential link of HDL and urticaria.

### 4.4. HDL in Angioedema

Angioedema, in the absence of urticaria, is a rare condition that manifests itself by sudden, localized, non-pitting, erythematous, or skin-colored swelling of certain body parts, including the skin, mucous membranes, or both, the upper respiratory and intestinal epithelial linings [234]. Heat and pain comprise additional symptoms of the skin, although they are hardly accompanied by itching, desquamation, or staining of the skin [234]. When present, angioedema should be diagnosed with caution, since alternative diagnoses, including acquired angioedema, hereditary angioedema, or angioedema that is associated with angiotensin-converting enzyme inhibitors, all comprising life-threatening conditions, might also be true [227]. It can be further classified to idiopathic, histaminergic, hereditary type I, hereditary type II, and hereditary with normal C1 inhibitor, acquired and angiotensin-converting enzyme (ACE) inhibitor-induced [227]. Angioedema results from the release of vasoactive mediators, which increase the vascular permeability in the skin and submucosa, leading to plasma vascular leakage and a resulting edema, which can be attributed either to bradykinin- or to histamine-mediated mechanisms [227]. The exact pathophysiology, diagnosis, and treatment options have been described elsewhere [227,234], and they are not in the scope of this review.

Several different studies have determined the serum levels of HDL-cholesterol in angioedema patients, however currently no literature on potential HDL-associated compositional alterations in angioedema is available. A study conducted by Sloane et al. evaluating the potential side effects of long-term stanozolol therapy in hereditary angioedema patients has revealed reduced HDL-cholesterol levels after stanozolol treatment is some of the patients [235]. Other studies, evaluating possible adverse effects of danazol treatment, revealed significantly lower levels of serum HDL-cholesterol [236,237] and apoA-I in danazol treated patients when compared to control groups (either untreated patients or patients without long-term danazol treatment), as well as a higher risk of abnormally low HDL-cholesterol levels in danazol treated patients, indicating that long-term use of this drug is associated with increased early atherosclerosis risk [236]. A similar study by Birjmohun et al., which evaluated the effects of short- and long-term danazol treatment, revealed decreased apoA-I and HDL-cholesterol levels in short-term treated patients in comparison to the baseline measures, while long-term treatment did not adversely affect HDL-cholesterol concentration and apolipoproteins between patients and controls [36]. A more recent study by Nebenführer et al. revealed that danazol treated patients suffering from hereditary angioedema with C1 inhibitor deficiency had higher cardiovascular risk, as evaluated by the high body mass index and LDL/HDL ratio, in comparison to healthy controls [238].

Currently, information on functionality of HDL-associated enzymes in angioedema patients is only available by the study of Birjmohun et al., which evaluated the effects of short- and long-term danazol treatment in hereditary angioedema patients. This study revealed no adverse effects of short- and long-term danazol treatment on PON-1, PLTP, and CETP activities along with CETP mass between patients and controls. However, a trend towards decreased LCAT activity was observed in the long-term, although unaltered in the short-term danazol treated patients [36]. Further studies in larger cohorts are necessary in order to confirm the observed effects and understand the possible pathophysiological role of HDL in angioedema.

## 5. Conclusions

**Figure 2:**
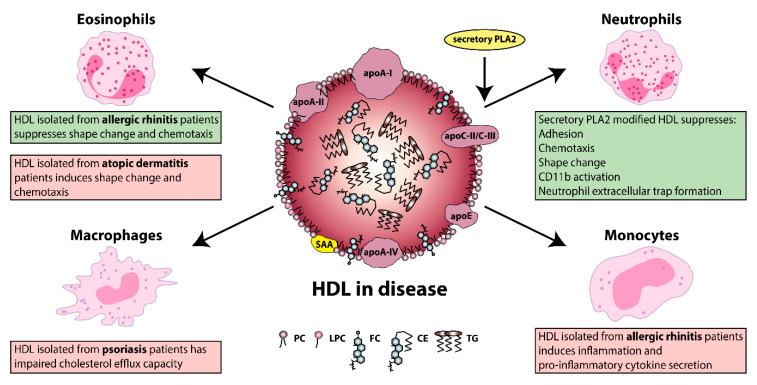
Overview of immunomodulating functions of HDL on different allergic and skin diseases. HDL differently modulates eosinophil effector responses in allergic rhinitis [29] and atopic dermatitis [35]. In psoriasis, HDL showed impaired HDL-cholesterol efflux capacity [30,32,33,34], while in allergic rhinitis HDL induced nuclear factor-κB (NF-κB) expression and pro-inflammatory cytokine secretion of monocytes [29]. Secretory phospholipase A2 (PLA2)-modified HDL prevented agonist-induced neutrophil activation, including shape change, cluster of differentiation (CD) 11b activation, neutrophil extracellular trap (NET) formation, adhesion under flow and migration of neutrophils [242]. Abbreviations represent: apoA-I, apolipoprotein A-I; apoA-II, apolipoprotein A-II; apoA-IV, apolipoprotein A-IV; apoC-II, apolipoprotein C-II; apoC-III, apolipoprotein C-III; apoE, apolipoprotein E; CD, cluster of differentiation; CE, cholesteryl ester; FC, free cholesterol; HDL, high-density lipoprotein; LPC, lyso-phosphatidylcholine; PC, phosphatidylcholine; PLA2, phospholipase A2; SAA, serum amyloid A; TG, triglyceride. Rectangle shadings represent: green, beneficial effect of HDL; red, detrimental effect of HDL.

From an evolutionary point of view, lipoproteins display important functions in many aspects of immunity. Of all lipoproteins, HDL has the highest affinity for binding and neutralizing pathogen-associated lipids (e.g., LPS and lipoteichoic acid) [41,239], which mediate excessive immune activation in bacterial infections [41,240,241]. Research into the composition, distribution, and functionality of HDL particles in allergic and skin diseases has begun to attract attention, with several groups demonstrating changes in the composition and function of HDL. Figure 2 summarizes the functional alterations of HDL on its immunomodulating abilities, due to a pathological background.

Of particular interest, not only a loss in function but also a gain in function regarding HDL properties is observed due to a specific pathological background. For instance, eosinophil effector responses are being effectively suppressed by HDL derived from allergic rhinitis patients; however atopic dermatitis-HDL induces rather the opposite effect [29,35]. These changes in the HDL functionality are at least partially explained by disease specific changes in HDL composition. HDL that is isolated from patients with allergic rhinitis contains elevated levels of lyso-phosphatidylcholine and apoC-III, whereas the opposite is seen in HDL from patients with atopic dermatitis.

A major weakness in HDL research is that HDL-cholesterol levels vary widely between different studies of the same disease background, which is possibly due to different study design, disease duration, or the presence of concomitant diseases, making it difficult to draw firm conclusions. However, the results of several studies provide compelling evidence that allergies and skin diseases significantly affect the composition and metabolism of HDL, which, in turn, could have a significant impact on disease progression and the risk of infection and cardiovascular disease.

HDL particles in allergic and inflammatory skin diseases have an altered composition, which results in an altered functionality; however, these changes are not consistent for different pathological backgrounds. Currently, there are no tests available for measuring the composition, function, and inflammatory properties of HDL in clinical practice. It is not clear to what extent inflammatory-HDL alterations are a driving force or only a biomarker of the disease. Future studies are needed in order to demonstrate causality.

ApoA-I, which is the major protein component of HDL particles, but also apoA-IV and certain HDL-associated lyso-phospholipids, are endogenous, anti-inflammatory mediators that potently suppress effector cell functions in eosinophils and neutrophils and show a variety of positive effects. Thus, exogenously applied apolipoproteins may represent a novel pharmacological approach for the treatment of allergic inflammation and inflammatory skin diseases. It remains to be seen whether these concepts can be translated into new therapeutic interventions for allergies and skin diseases.

## Abbreviations

ABC	ATP-binding cassette
ACE	angiotensin-converting enzyme
AMPK	adenosine monophosphate-activated protein kinase
apo	apolipoprotein
CD	cluster of differentiation
CE	cholesteryl ester
CETP	cholesteryl ester transfer protein
EL	endothelial lipase
FC	free cholesterol
FEV_1_	forced expiratory volume in one second
FVC	forced vital capacity
HDL	high-density lipoprotein
HL	hepatic lipase
IgE	immunoglobulin E
IL	interleukin
JAK	janus kinase
LCAT	lecithin-cholesterol acyltransferase
LDL	low-density lipoprotein
LDLR	low-density lipoprotein receptor
LPC	lyso-phosphatidylcholine
LPL	lipoprotein lipase
Lp-PLA2	lipoprotein-associated phospholipase A2
LPS	lipopolysaccharide
NET	neutrophil extracellular trap
NF-κB	nuclear factor-κB
PAF	platelet-activating factor
PAF-AH	platelet-activating factor-acetylhydrolase
PC	phosphatidylcholine
PLA2	phospholipase A2
PLTP	phospholipid transfer protein
PON	paraoxonase
PUVA	psoralen and ultraviolet A
RCT	reverse cholesterol transport
SAA	serum amyloid A
SR-BI	scavenger receptor class B type I
TG	triglyceride
TLRs	toll-like receptors
TNF	tumor necrosis factor
UVB	ultraviolet B
VLDL	very low-density lipoprotein

## References

[1] Karimkhani C., Dellavalle R.P., Coffeng L.E., Flohr C., Hay R.J., Langan S.M., Nsoesie E.O., Ferrari A.J., Erskine H.E., Silverberg J.I. (2017). Global skin disease morbidity and mortality an update from the global burden of disease study 2013. JAMA Dermatol..

[2] Pawankar R. (2014). Allergic diseases and asthma: A global public health concern and a call to action. World Allergy Organ. J..

[3] Yu B., Wang S., Peng D., Zhao S. (2010). HDL and immunomodulation: An emerging role of HDL against atherosclerosis. Immunol. Cell Biol..

[4] Catapano A.L., Pirillo A., Bonacina F., Norata G.D. (2014). HDL in innate and adaptive immunity. Cardiovasc. Res..

[5] Fessler M.B. (2012). Next stop for HDL: The lung. Clin. Exp. Allergy.

[6] Shenoy C., Shenoy M.M., Rao G.K. (2015). Dyslipidemia in dermatological disorders. N. Am. J. Med. Sci..

[7] Di Angelantonio E., Sarwar N., Perry P., Kaptoge S., Ray K.K., Thompson A., Wood A.M., Lewington S., Sattar N., Packard C.J. (2009). Major lipids, apolipoproteins, and risk of vascular disease. JAMA J. Am. Med. Assoc..

[8] Gordon T., Castelli W., Hjortland M.C. (1977). High density lipoprotein as a protective factor against coronary heart disease: The Framingham Study. Am. J. Med..

[9] Wilson P.W.F., Abbott R.D., Castelli W.P. (1988). High density lipoprotein cholesterol and mortality. The Framingham heart study. Arteriosclerosis.

[10] Gordon D.J., Probstfield J.L., Garrison R.J., Neaton J.D., Castelli W.P., Knoke J.D., Jacobs D.R., Bangdiwala S., Tyroler H.A. (1989). High-density lipoprotein cholesterol and cardiovascular disease. Four prospective American studies. Circulation.

[11] Karalis I., Jukema J.W. (2018). HDL Mimetics Infusion and Regression of Atherosclerosis: Is It Still Considered a Valid Therapeutic Option?. Curr. Cardiol. Rep..

[12] Nicholls S.J., Andrews J., Kastelein J.J.P., Merkely B., Nissen S.E., Ray K.K., Schwartz G.G., Worthley S.G., Keyserling C., Dasseux J.L. (2018). Effect of serial infusions of CER-001, a pre-β High-density lipoprotein mimetic, on coronary atherosclerosis in patients following acute coronary syndromes in the CER-001 atherosclerosis regression acute coronary syndrome trial: A randomized clinical trial. JAMA Cardiol..

[13] Armitage J., Holmes M.V., Preiss D. (2019). Cholesteryl Ester Transfer Protein Inhibition for Preventing Cardiovascular Events: JACC Review Topic of the Week. J. Am. Coll. Cardiol..

[14] Holmes M.V., Asselbergs F.W., Palmer T.M., Drenos F., Lanktree M.B., Nelson C.P., Dale C.E., Padmanabhan S., Finan C., Swerdlow D.I. (2015). Mendelian randomization of blood lipids for coronary heart disease. Eur. Heart J..

[15] Norata G.D., Pirillo A., Ammirati E., Catapano A.L. (2012). Emerging role of high density lipoproteins as a player in the immune system. Atherosclerosis.

[16] Madsen C.M., Varbo A., Nordestgaard B.G. (2019). Low HDL Cholesterol and high risk of autoimmune disease: Two population-based cohort studies including 117341 individuals. Clin. Chem..

[17] Davidson W.S., Thompson T.B. (2007). The structure of apolipoprotein A-I in high density lipoproteins. J. Biol. Chem..

[18] Javaheri A., Rader D.J. (2014). Apolipoprotein A-I and cholesterol efflux: The good, the bad, and the modified. Circ. Res..

[19] Sorci-Thomas M.G., Thomas M.J. (2012). High density lipoprotein biogenesis, cholesterol efflux, and immune cell function. Arterioscler. Thromb. Vasc. Biol..

[20] Fessler M.B., Parks J.S. (2011). Intracellular Lipid Flux and Membrane Microdomains as Organizing Principles in Inflammatory Cell Signaling. J. Immunol..

[21] Varshney P., Yadav V., Saini N. (2016). Lipid rafts in immune signalling: Current progress and future perspective. Immunology.

[22] Wang S., Yuan S., Peng D., Zhao S. (2012). HDL and ApoA-I inhibit antigen presentation-mediated T cell activation by disrupting lipid rafts in antigen presenting cells. Atherosclerosis.

[23] Law S.-H., Chan M.-L., Marathe G.K., Parveen F., Chen C.-H., Ke L.-Y. (2019). An Updated Review of Lysophosphatidylcholine Metabolism in Human Diseases. Int. J. Mol. Sci..

[24] Carneiro A.B., Iaciura B.M.F., Nohara L.L., Lopes C.D., Veas E.M.C., Mariano V.S., Bozza P.T., Lopes U.G., Atella G.C., Almeida I.C. (2013). Lysophosphatidylcholine Triggers TLR2- and TLR4-Mediated Signaling Pathways but Counteracts LPS-Induced NO Synthesis in Peritoneal Macrophages by Inhibiting NF-κB Translocation and MAPK/ERK Phosphorylation. PLoS ONE.

[25] Miller L.S., Modlin R.L. (2007). Toll-like receptors in the skin. Semin. Immunopathol..

[26] Kawasaki T., Kawai T. (2014). Toll-like receptor signaling pathways. Front. Immunol..

[27] Kang S.S.W., Kauls L.S., Gaspari A.A. (2006). Toll-like receptors: Applications to dermatologic disease. J. Am. Acad. Dermatol..

[28] McInturff J.E., Modlin R.L., Kim J. (2005). The role of toll-like receptors in the pathogenesis and treatment of dermatological disease. J. Investig. Dermatol..

[29] Trakaki A., Sturm G.J., Pregartner G., Scharnagl H., Eichmann T.O., Trieb M., Knuplez E., Holzer M., Stadler J.T., Heinemann A. (2019). Allergic rhinitis is associated with complex alterations in high-density lipoprotein composition and function. Biochim. Biophys. Acta Mol. Cell Biol. Lipids.

[30] Holzer M., Wolf P., Curcic S., Birner-Gruenberger R., Weger W., Inzinger M., El-Gamal D., Wadsack C., Heinemann A., Marsche G. (2012). Psoriasis alters HDL composition and cholesterol efflux capacity. J. Lipid Res..

[31] Yu Y., Sheth N., Krishnamoorthy P., Saboury B., Raper A., Baer A., Ochotony R., Doveikis J., Derohannessian S., Van Voorhees A.S. (2012). Aortic vascular inflammation in psoriasis is associated with HDL particle size and concentration: A pilot study. Am. J. Cardiovasc. Dis..

[32] Tom W.L., Playford M.P., Admani S., Natarajan B., Joshi A.A., Eichenfield L.F., Mehta N.N. (2016). Characterization of lipoprotein composition and function in pediatric psoriasis reveals a more atherogenic profile. J. Investig. Dermatol..

[33] Holzer M., Wolf P., Inzinger M., Trieb M., Curcic S., Pasterk L., Weger W., Heinemann A., Marsche G. (2014). Anti-psoriatic therapy recovers high-density lipoprotein composition and function. J. Investig. Dermatol..

[34] Mehta N.N., Li R., Krishnamoorthy P., Yu Y.D., Farver W., Rodrigues A., Raper A., Wilcox M., Baer A., DerOhannesian S. (2012). Abnormal lipoprotein particles and cholesterol efflux capacity in patients with psoriasis. Atherosclerosis.

[35] Trieb M., Wolf P., Knuplez E., Weger W., Schuster C., Peinhaupt M., Holzer M., Trakaki A., Eichmann T., Lass A. (2019). Abnormal composition and function of high-density lipoproteins in atopic dermatitis patients. Allergy Eur. J. Allergy Clin. Immunol..

[36] Birjmohun R.S., Kees Hovingh G., Stroes E.S.G., Hofstra J.J., Dallinga-Thie G.M., Meijers J.C.M., Kastelein J.J.P., Levi M. (2008). Effects of short-term and long-term danazol treatment on lipoproteins, coagulation, and progression of atherosclerosis: Two clinical trials in healthy volunteers and patients with hereditary angioedema. Clin. Ther..

[37] Zannis V.I., Chroni A., Kypreos K.E., Kan H.Y., Cesar T.B., Zanni E.E., Kardassis D. (2004). Probing the pathways of chylomicron and HDL metabolism using adenovirus-mediated gene transfer. Curr. Opin. Lipidol..

[38] Zannis V.I., Cole F.S., Jackson C.L., Kurnit D.M., Karathanasis S.K. (1985). Distribution of Apolipoprotein A-I, C-II, C-III, and E mRNA in Fetal Human Tissues. Time-Dependent Induction of Apolipoprotein E mRNA by Cultures of Human Monocyte-Macrophages. Biochemistry.

[39] Zannis V.I., Fotakis P., Koukos G., Kardassis D., Ehnholm C., Jauhiainen M., Chroni A., Von Eckardstein A., Kardassis D. (2015). Hdl biogenesis, remodeling, and catabolism. High Density Lipoproteins.

[40] Mackness M.I., Durrington P.N. (1995). HDL, its enzymes and its potential to influence lipid peroxidation. Atherosclerosis.

[41] Meilhac O., Tanaka S., Couret D. (2020). High-density lipoproteins are bug scavengers. Biomolecules.

[42] Barter P.J., Brewer H.B., Chapman M.J., Hennekens C.H., Rader D.J., Tall A.R. (2003). Cholesteryl Ester Transfer Protein. Arterioscler. Thromb. Vasc. Biol..

[43] Zhang M., Zhai X., Li J., Albers J.J., Vuletic S., Ren G. (2018). Structural basis of the lipid transfer mechanism of phospholipid transfer protein (PLTP). Biochim. Biophys. Acta Mol. Cell Biol. Lipids.

[44] Albers J.J., Cheung M.C. (2004). Emerging roles for phospholipid transfer protein in lipid and lipoprotein metabolism. Curr. Opin. Lipidol..

[45] Von Eckardstein A., Kardassis D. (2015). High Density Lipoproteins: From Biological Understanding to Clinical Exploitation.

[46] Lewis G.F., Rader D.J. (2005). New insights into the regulation of HDL metabolism and reverse cholesterol transport. Circ. Res..

[47] Yvan-Charvet L., Wang N., Tall A.R. (2010). The role of HDL, ABCA1, and ABCG1 transporters in cholesterol efflux and immune responses. Arterioscler. Thromb. Vasc. Biol..

[48] Maugeais C., Tietge U.J.F., Broedl U.C., Marchadier D., Cain W., McCoy M.G., Lund-Katz S., Glick J.M., Rader D.J. (2003). Dose-Dependent Acceleration of High-Density Lipoprotein Catabolism by Endothelial Lipase. Circulation.

[49] Santamarina-Fojo S., González-Navarro H., Freeman L., Wagner E., Nong Z. (2004). Hepatic lipase, lipoprotein metabolism, and atherogenesis. Arterioscler. Thromb. Vasc. Biol..

[50] Acton S., Rigotti A., Landschulz K.T., Xu S., Hobbs H.H., Kriegert M. (1996). Identification of scavenger receptor SR-BI as a high density lipoprotein receptor. Science (80-).

[51] Krieger M. (1999). Charting the Fate of the “Good Cholesterol”: Identification and Characterization of the High-Density Lipoprotein Receptor SR-BI. Annu. Rev. Biochem..

[52] Pagler T.A., Rhode S., Neuhofer A., Laggner H., Strobl W., Hinterndorfer C., Volf I., Pavelka M., Eckhardt E.R.M., Van Der Westhuyzen D.R. (2006). SR-BI-mediated high density lipoprotein (HDL) endocytosis leads to HDL resecretion facilitating cholesterol efflux. J. Biol. Chem..

[53] Kontush A., Lindahl M., Lhomme M., Calabresi L., Chapman M.J., Davidson W.S. (2015). Structure of HDL: Particle subclasses and molecular components. Handbook of Experimental Pharmacology.

[54] Camont L., Chapman M.J., Kontush A. (2011). Biological activities of HDL subpopulations and their relevance to cardiovascular disease. Trends Mol. Med..

[55] Vaisar T. (2012). Proteomics investigations of HDL: Challenges and promise. Curr. Vasc. Pharmacol..

[56] Wilkins J.T., Seckler H.S. (2019). HDL modification: Recent developments and their relevance to atherosclerotic cardiovascular disease. Curr. Opin. Lipidol..

[57] Davidson W.S., Silva R.A.G.D., Chantepie S., Lagor W.R., Chapman M.J., Kontush A. (2009). Proteomic analysis of defined hdl subpopulations reveals particle-specific protein clusters: Relevance to antioxidative function. Arterioscler. Thromb. Vasc. Biol..

[58] Holzer M., Kern S., Birner-Grünberger R., Curcic S., Heinemann A., Marsche G. (2016). Refined purification strategy for reliable proteomic profiling of HDL 2/3: Impact on proteomic complexity. Sci. Rep..

[59] Shao B., Heinecke J.W. (2018). Quantifying HDL proteins by mass spectrometry: How many proteins are there and what are their functions?. Expert Rev. Proteom..

[60] Davidson W.S. HDL Proteome Watch. The Davidson & Shah Lab Website. https://homepages.uc.edu/~davidswm/HDLproteome.html.

[61] Wiesner P., Leidl K., Boettcher A., Schmitz G., Liebisch G. (2009). Lipid profiling of FPLC-separated lipoprotein fractions by electrospray ionization tandem mass spectrometry. J. Lipid Res..

[62] Scherer M., Böttcher A., Liebisch G. (2011). Lipid profiling of lipoproteins by electrospray ionization tandem mass spectrometry. Biochim. Biophys. Acta Mol. Cell Biol. Lipids.

[63] Vuorela T., Catte A., Niemelä P.S., Hall A., Hyvönen M.T., Marrink S.J., Karttunen M., Vattulainen I. (2010). Role of Lipids in Spheroidal High Density Lipoproteins. PLoS Comput. Biol..

[64] Frame N.M., Gursky O. (2016). Structure of serum amyloid A suggests a mechanism for selective lipoprotein binding and functions: SAA as a hub in macromolecular interaction networks. FEBS Lett..

[65] Ferretti G., Bacchetti T., Moroni C., Savino S., Liuzzi A., Balzola F., Bicchiega V. (2005). Paraoxonase Activity in High-Density Lipoproteins: A Comparison between Healthy and Obese Females. J. Clin. Endocrinol. Metab..

[66] Stafforini D.M., McIntyre T.M., Carter M.E., Prescott S.M. (1987). Human plasma platelet-activating factor acetylhydrolase. Association with lipoprotein particles and role in the degradation of platelet-activating factor. J. Biol. Chem..

[67] Snyder F. (1995). Platelet-activating factor and its analogs: Metabolic pathways and related intracellular processes. Biochim. Biophys. Acta (BBA)/Lipids Lipid Metab..

[68] Stafforini D.M., McIntyre T.M., Zimmerman G.A., Prescott S.M. (1997). Platelet-activating factor acetylhydrolases. J. Biol. Chem..

[69] Chowaniec Z., Skoczyńska A. (2018). Plasma lipid transfer proteins: The role of PLTP and CETP in atherogenesis. Adv. Clin. Exp. Med..

[70] Von Eckardstein A. (2012). Tachometer for Reverse Cholesterol Transport?. J. Am. Heart Assoc..

[71] Mukherjee A.B., Zhang Z. (2011). Allergic asthma: Influence of genetic and environmental factors. J. Biol. Chem..

[72] Idani E., Raji H., Madadizadeh F., Cheraghian B., Haddadzadeh Shoshtari M., Dastoorpoor M. (2019). Prevalence of asthma and other allergic conditions in adults in Khuzestan, southwest Iran, 2018. BMC Public Health.

[73] Locksley R.M. (2010). Asthma and Allergic Inflammation. Cell.

[74] Quirt J., Hildebrand K.J., Mazza J., Noya F., Kim H. (2018). Asthma. Allergy Asthma Clin. Immunol..

[75] Fenger R.V., Gonzalez-Quintela A., Linneberg A., Husemoen L.L.N., Thuesen B.H., Aadahl M., Vidal C., Skaaby T., Sainz J.C., Calvo E. (2013). The relationship of serum triglycerides, serum HDL, and obesity to the risk of wheezing in 85,555 adults. Respir. Med..

[76] Schäfer T., Ruhdorfer S., Weigl L., Wessner D., Heinrich J., Döring A., Wichmann H.E., Ring J. (2003). Intake of unsaturated fatty acids and HDL cholesterol levels are associated with manifestations of atopy in adults. Clin. Exp. Allergy.

[77] Rastogi D., Jung M., Strizich G., Shaw P.A., Davis S.M., Klein O.L., Penedo F.J., Ries A.L., Daviglus M.L., Moreiras J.J. (2017). Association of systemic inflammation, adiposity, and metabolic dysregulation with asthma burden among Hispanic adults. Respir. Med..

[78] Sobko E.A., Solovyeva I.A., Demko I.V., Kraposhina A.Y., Ishchenko O.P., Razzakova N.M., Egorov S.A., Vtyurina S.S., Prugova V.L. (2016). Functional and laboratory characteristics in the concomitance of asthma and obesity at a young age. Ter. Arkh..

[79] Shenoi A., Kumar L., Sarihyan S., Gangully N. (1992). High density lipoprotein cholesterol and total cholesterol in children with asthma and allergic rhinitis. Acta Paediatr..

[80] Fessler M.B., Massing M.W., Spruell B., Jaramillo R., Draper D.W., Madenspacher J.H., Arbes S.J., Calatroni A., Zeldin D.C. (2009). Novel relationship of serum cholesterol with asthma and wheeze in the United States. J. Allergy Clin. Immunol..

[81] Enright P.L., Ward B.J., Tracy R.P., Lasser E.C. (1996). Asthma and its association with cardiovascular disease in the elderly. J. Asthma.

[82] Park S., Choi N.K., Kim S., Lee C.H. (2018). The relationship between metabolic syndrome and asthma in the elderly. Sci. Rep..

[83] Picado C., Deulofeu R., Lleonart R., Agustí M., Casals E., Quintó L., Mullol J. (1999). Lipid and protein metabolism in asthma. Effects of diet and corticosteroid therapy. Allergy Eur. J. Allergy Clin. Immunol..

[84] Erel F., Gulec M., Kartal O., Caliskaner Z., Ozturk S., Yaman H., Kurt Y., Gocgeldi E., Ors F., Karaayvaz M. (2007). Serum Leptin Levels and Lipid Profiles in Patients with Allergic Rhinitis and Mild Asthma. Allergol. Immunopathol. (Madr.).

[85] Lu M., Wu B., Qiao R., Gu H., Din Y., Dong X. (2019). No associations between serum lipid levels or HOMA-IR and asthma in children and adolescents: A NHANES analysis. JCRPE J. Clin. Res. Pediatr. Endocrinol..

[86] Fang L.J., Huang C.S., Liu Y.C., Su Y.M., Wan K.S. (2016). The lipid profile in obese asthmatic children compared to non-obese asthmatic children. Allergol. Immunopathol. (Madr.).

[87] Cottrell L., Neal W.A., Ice C., Perez M.K., Piedimonte G. (2011). Metabolic abnormalities in children with asthma. Am. J. Respir. Crit. Care Med..

[88] Yiallouros P.K., Savva S.C., Kolokotroni O., Dima K., Zerva A., Kouis P., Bousquet J., Middleton N. (2014). Asthma: The Role of Low High-Density-Lipoprotein Cholesterol in Childhood and Adolescence. Int. Arch. Allergy Immunol..

[89] Yiallouros P.K., Savva S.C., Kolokotroni O., Behbod B., Zeniou M., Economou M., Chadjigeorgiou C., Kourides Y.A., Tornaritis M.J., Lamnisos D. (2012). Low serum high-density lipoprotein cholesterol in childhood is associated with adolescent asthma. Clin. Exp. Allergy.

[90] Cakmak A., Zeyrek D., Atas A., Selek S., Erel O. (2009). Oxidative status and paraoxonase activity in children with asthma. Clin. Investig. Med..

[91] Gulcan E., Bulut I., Toker A., Gulcan A. (2009). Evaluation of glucose tolerance status in patients with asthma bronchiale. J. Asthma.

[92] Scichilone N., Rizzo M., Benfante A., Catania R., Giglio R.V., Nikolic D., Montalto G., Bellia V. (2013). Serum low density lipoprotein subclasses in asthma. Respir. Med..

[93] Ekmekci O.B., Donma O., Ekmekci H., Yildirim N., Uysal O., Sardogan E., Demirel H., Demir T. (2006). Plasma paraoxonase activities, lipoprotein oxidation, and trace element interaction in asthmatic patients. Biol. Trace Elem. Res..

[94] Dominic J.C., Yuri Agrawal P.A.C. (2002). Lipids and Pulmonary Function in the Third National Health and Nutrition Examination Survey. Am. J. Epidemiol..

[95] Peng J., Huang Y. (2017). Meta-analysis of the association between asthma and serum levels of high-density lipoprotein cholesterol and low-density lipoprotein cholesterol. Ann. Allergy Asthma Immunol..

[96] Barochia A.V., Kaler M., Cuento R.A., Gordon E.M., Weir N.A., Sampson M., Fontana J.R., MacDonald S., Moss J., Manganiello V. (2015). Serum Apolipoprotein A-I and large high-density lipoprotein particles are positively correlated with fev1 in atopic asthma. Am. J. Respir. Crit. Care Med..

[97] Barochia A.V., Kaler M., Cuento R., Gordon E.M., Theard P., Figueroa D., Weir N., Sampson M., Remaley A.T. (2016). Serum High Density Lipoprotein (hdl) Cholesterol And Large Hdl Particles Are Negatively Correlated With Blood Eosinophils In Atopic Asthma. Am. J. Respir. Crit. Care Med..

[98] Barochia A.V., Gordon E.M., Kaler M., Cuento R.A., Theard P., Figueroa D.M., Yao X., Weir N.A., Sampson M.L., Stylianou M. (2017). High density lipoproteins and type 2 inflammatory biomarkers are negatively correlated in atopic asthmatics. J. Lipid Res..

[99] Rastogi D., Fraser S., Oh J., Huber A.M., Schulman Y., Bhagtani R.H., Khan Z.S., Tesfa L., Hall C.B., Macian F. (2015). Inflammation, metabolic dysregulation, and pulmonary function among obese urban adolescents with asthma. Am. J. Respir. Crit. Care Med..

[100] Ouyang F., Kumar R., Pongracic J., Story R.E., Liu X., Wang B., Xing H., Liu X., Li Z., Zhang W. (2009). Adiposity, serum lipid levels, and allergic sensitization in Chinese men and women. J. Allergy Clin. Immunol..

[101] Su X., Ren Y., Li M., Zhao X., Kong L., Kang J. (2018). Association between lipid profile and the prevalence of asthma: A meta-analysis and systemic review. Curr. Med. Res. Opin..

[102] Rasmussen F., Hancox R.J., Nair P., Hansen H.S., Siersted H.C., Nybo M. (2013). Associations between airway hyperresponsiveness, obesity and lipoproteins in a longitudinal cohort. Clin. Respir. J..

[103] Park J.H., Mun S., Choi D.P., Lee J.Y., Kim H.C. (2017). Association between high-density lipoprotein cholesterol level and pulmonary function in healthy Korean adolescents: The JS high school study. BMC Pulm. Med..

[104] Park S.W., Lee E.H., Lee E.J., Kim H.J., Bae D.J., Han S., Kim D., Jang A.S., Uh S.T., Kim Y.H. (2013). Apolipoprotein A1 potentiates lipoxin A4 synthesis and recovery of allergen-induced disrupted tight junctions in the airway epithelium. Clin. Exp. Allergy.

[105] Yao X., Gordon E.M., Barochia A.V., Remaley A.T., Levine S.J. (2016). The A’s Have It: Developing Apolipoprotein A-I Mimetic Peptides Into a Novel Treatment for Asthma. Chest.

[106] Thorne P.S., Mendy A., Metwali N., Salo P., Co C., Jaramillo R., Rose K.M., Zeldin D.C. (2015). Endotoxin exposure: Predictors and prevalence of associated asthma outcomes in the United States. Am. J. Respir. Crit. Care Med..

[107] Thorne P.S., Kulhánková K., Yin M., Cohn R., Arbes S.J., Zeldin D.C. (2005). Endotoxin exposure is a risk factor for asthma: The national survey of endotoxin in United States housing. Am. J. Respir. Crit. Care Med..

[108] Kim K.D., Lim H.Y., Lee H.G., Yoon D.Y., Choe Y.K., Choi I., Paik S.G., Kim Y.S., Yang Y., Lim J.S. (2005). Apolipoprotein A-I induces IL-10 and PGE2 production in human monocytes and inhibits dendritic cell differentiation and maturation. Biochem. Biophys. Res. Commun..

[109] Tiniakou I., Drakos E., Sinatkas V., Van Eck M., Zannis V.I., Boumpas D., Verginis P., Kardassis D. (2015). High-Density Lipoprotein Attenuates Th1 and Th17 Autoimmune Responses by Modulating Dendritic Cell Maturation and Function. J. Immunol..

[110] Nicholas B.L., Skipp P., Barton S., Singh D., Bagmane D., Mould R., Angco G., Ward J., Guha-Niyogi B., Wilson S. (2010). Identification of lipocalin and apolipoprotein A1 as biomarkers of chronic obstructive pulmonary disease. Am. J. Respir. Crit. Care Med..

[111] Kuczia P., Mastalerz L., Potaczek D.P., Cybulska A., Zareba L., Bazan-Socha S., Undas A. (2019). Increased activity of lipoprotein-associated phospholipase A2 in non-severe asthma. Allergol. Int..

[112] Grandel K.E., Wardlow M.L., Farr R.S. (1985). Platelet activating factor in sputum of patients with asthma and COPD. J. Allergy Clin. Immunol..

[113] Stenton S.C., Court E.N., Kingston W.P., Goadby P., Kelly C.A., Duddridge M., Ward C., Hendrick D.J., Walters E.H. (1990). Platelet-activating factor in bronchoalveolar lavage fluid from asthmatic subjects. Eur. Respir. J..

[114] Kurosawa M., Yamashita T., Kurimoto F. (1994). Increased levels of blood platelet-activating factor in bronchial asthmatic patients with active symptoms. Allergy.

[115] Tsukioka K., Matsuzaki M., Nakamata M., Kayahara H. (1996). Increased Plasma Level of Platelet-Activating Factor (PAF) and Decreased Serum PAF Acetylhydrolase (PAFAH) Activity in Adults With Bronchial Asthma. J. Investig. Allergol. Clin. Immunol..

[116] Hsieh K.H., Ng C.K. (1993). Increased plasma platelet-activating factor in children with acute asthmatic attacks and decreased in vivo and in vitro production of platelet-activating factor after immunotherapy. J. Allergy Clin. Immunol..

[117] Chan-Yeung M., Lam S., Chan H., Tse K.S., Salari H. (1991). The release of platelet-activating factor into plasma during allergen-induced bronchoconstriction. J. Allergy Clin. Immunol..

[118] Shirasaki H., Nishikawa M., Adcock I.M., Mak J.C., Sakamoto T., Shimizu T., Barnes P.J. (1994). Expression of platelet-activating factor receptor mRNA in human and guinea pig lung. Am. J. Respir. Cell Mol. Biol..

[119] Wang W., Xu H., Shi Y., Nandedkar S., Zhang H., Gao H., Feroah T., Weihrauch D., Schulte M.L., Jones D.W. (2010). Genetic deletion of apolipoprotein A-I increases airway hyperresponsiveness, inflammation, and collagen deposition in the lung. J. Lipid Res..

[120] Yao X., Gordon E.M., Figueroa D.M., Barochia A.V., Levine S.J. (2016). Emerging roles of apolipoprotein e and apolipoprotein A-I in the pathogenesis and treatment of lung disease. Am. J. Respir. Cell Mol. Biol..

[121] Dai C., Yao X., Keeran K.J., Zywicke G.J., Qu X., Yu Z.X., Dagur P.K., McCoy J.P., Remaley A.T., Levine S.J. (2012). Apolipoprotein A-I attenuates ovalbumin-induced neutrophilic airway inflammation via a granulocyte colony-stimulating factor-dependent mechanism. Am. J. Respir. Cell Mol. Biol..

[122] Yao X., Vitek M.P., Remaley A.T., Levine S.J. (2012). Apolipoprotein mimetic peptides: A new approach for the treatment of asthma. Front. Pharmacol..

[123] Nandedkar S.D., Weihrauch D., Xu H., Shi Y., Feroah T., Hutchins W., Rickaby D.A., Duzgunes N., Hillery C.A., Konduri K.S. (2011). D-4F, an apoA-1 mimetic, decreases airway hyperresponsiveness, inflammation, and oxidative stress in a murine model of asthma. J. Lipid Res..

[124] Eifan A.O., Durham S.R. (2016). Pathogenesis of rhinitis. Clin. Exp. Allergy.

[125] Small P., Keith P.K., Kim H. (2018). Allergic rhinitis. Allergy Asthma Clin. Immunol..

[126] Vinding R.K., Stokholm J., Chawes B.L.K., Bisgaard H. (2016). Blood lipid levels associate with childhood asthma, airway obstruction, bronchial hyperresponsiveness, and aeroallergen sensitization. J. Allergy Clin. Immunol..

[127] Tomazic P.V., Birner-Gruenberger R., Leitner A., Obrist B., Spoerk S., Lang-Loidolt D. (2014). Nasal mucus proteomic changes reflect altered immune responses and epithelial permeability in patients with allergic rhinitis. J. Allergy Clin. Immunol..

[128] Tomazic P.V., Birner-Gruenberger R., Leitner A., Darnhofer B., Spoerk S., Lang-Loidolt D. (2015). Apolipoproteins have a potential role in nasal mucus of allergic rhinitis patients: A proteomic study. Laryngoscope.

[129] Tomazic P., Birner-Grünberger R., Britta O., Spörk S., Leitner A., Lang-Loidolt D. (2014). The (potential) role of apolipoproteins in nasal mucus of allergic rhinitis patients. Clin. Transl. Allergy.

[130] Kåredal M.H., Mortstedt H., Jeppsson M.C., Kronholm Diab K., Nielsen J., Jonsson B.A.G., Lindh C.H. (2010). Time-dependent proteomic iTRAQ analysis of nasal lavage of hairdressers challenged by persulfate. J. Proteome Res..

[131] Jaramillo R., Cohn R.D., Crockett P.W., Gowdy K.M., Zeldin D.C., Fessler M.B. (2013). Relation between objective measures of atopy and myocardial infarction in the United States. J. Allergy Clin. Immunol..

[132] Trasande L., Fiorino E.K., Attina T., Berger K., Goldring R., Chemtob C., Levy-Carrick N., Shao Y., Liu M., Urbina E. (2013). Associations of World Trade Center exposures with pulmonary and cardiometabolic outcomes among children seeking care for health concerns. Sci. Total Environ..

[133] Lee E.J., Hwang H.J., Jung C.M., Kim M.K., Kang M.S., Kim K.S. (2017). The relationship between chronic rhinosinusitis and metabolic syndrome. Am. J. Rhinol. Allergy.

[134] Roula D., Theiler A., Luschnig P., Sturm G.J., Tomazic P.V., Marsche G., Heinemann A., Sturm E.M. (2020). Apolipoprotein A-IV acts as an endogenous anti-inflammatory protein and is reduced in treatment-naïve allergic patients and allergen-challenged mice. Allergy.

[135] Makino Y., Noguchi E., Takahashi N., Matsumoto Y., Kubo S., Yamada T., Imoto Y., Ito Y., Osawa Y., Shibasaki M. (2010). Apolipoprotein A-IV is a candidate target molecule for the treatment of seasonal allergic rhinitis. J. Allergy Clin. Immunol..

[136] Chung Y.W., Cha J., Han S., Chen Y., Gucek M., Cho H.J., Nakahira K., Choi A.M.K., Ryu J.H., Yoon J.H. (2020). Apolipoprotein e and periostin are potential biomarkers of nasal mucosal inflammation a parallel approach of in vitro and in vivo secretomes. Am. J. Respir. Cell Mol. Biol..

[137] Dykewicz M.S., Hamilos D.L. (2010). Rhinitis and sinusitis. J. Allergy Clin. Immunol..

[138] Upton D.C., Welham N.V., Kuo J.S., Walker J.W., Pasic T.R. (2011). Chronic rhinosinusitis with nasal polyps: A proteomic analysis. Ann. Otol. Rhinol. Laryngol..

[139] Ozkaya E., Akduman H., Erenberk U., Demir A., Dundaroz M.R. (2013). Plasma paraoxonase activity and oxidative stress and their relationship to disease severity in children with allergic rhinitis. Am. J. Rhinol. Allergy.

[140] Fonacier L.S., Dreskin S.C., Leung D.Y.M., Mineola C., Denver A. (2010). Allergic skin diseases. J Allergy Clin Immunol..

[141] Williams H., Robertson C., Stewart A., Aït-Khaled N., Anabwani G., Anderson R., Asher I., Beasley R., Björkstén B., Burr M. (1999). Worldwide variations in the prevalence of symptoms of atopic eczema in the international study of asthma and allergies in childhood. J. Allergy Clin. Immunol..

[142] Bieber T. (2012). Atopic dermatitis 2.0: From the clinical phenotype to the molecular taxonomy and stratified medicine. Allergy Eur. J. Allergy Clin. Immunol..

[143] Garmhausen D., Hagemann T., Bieber T., Dimitriou I., Fimmers R., Diepgen T., Novak N. (2013). Characterization of different courses of atopic dermatitis in adolescent and adult patients. Allergy Eur. J. Allergy Clin. Immunol..

[144] Wu L.C., Hwang C.Y., Chung P.I., Hua T.C., Da Chen Y., Chu S.Y., Lee D.D., Chang Y.T., Wang W.J., Liu H.N. (2014). Autoimmune disease comorbidities in patients with atopic dermatitis: A nationwide case-control study in Taiwan. Pediatr. Allergy Immunol..

[145] Silverberg J.I., Greenland P. (2015). Eczema and cardiovascular risk factors in 2 US adult population studies. J. Allergy Clin. Immunol..

[146] Andersen Y.M.F., Egeberg A., Gislason G.H., Hansen P.R., Skov L., Thyssen J.P. (2016). Risk of myocardial infarction, ischemic stroke, and cardiovascular death in patients with atopic dermatitis. J. Allergy Clin. Immunol..

[147] Su V.Y.F., Chen T.J., Yeh C.M., Chou K.T., Hung M.H., Chu S.Y., Su K.C., Chang Y.S., Lin Y.H., Liu C.J. (2014). Atopic dermatitis and risk of ischemic stroke: A nationwide population-based study. Ann. Med..

[148] Silverberg J.I. (2015). Association between adult atopic dermatitis, cardiovascular disease, and increased heart attacks in three population-based studies. Allergy Eur. J. Allergy Clin. Immunol..

[149] Standl M., Tesch F., Baurecht H., Rodríguez E., Müller-Nurasyid M., Gieger C., Peters A., Wang-Sattler R., Prehn C., Adamski J. (2017). Association of Atopic Dermatitis with Cardiovascular Risk Factors and Diseases. J. Investig. Dermatol..

[150] Agón-Banzo P.J., Sanmartin R., García-Malinis A.J., Hernández-Martín Á., Puzo J., Doste D., Pardos C., Gilaberte Y. (2020). Body mass index and serum lipid profile: Association with atopic dermatitis in a paediatric population. Australas. J. Dermatol..

[151] Yamane Y., Moriyama K., Yasuda C., Miyata S., Aihara M., Ikezawa Z., Miyazaki K. (2009). New horny layer marker proteins for evaluating skin condition in atopic dermatitis. Int. Arch. Allergy Immunol..

[152] Liu F.T., Goodarzi H., Chen H.Y. (2011). IgE, mast cells, and eosinophils in atopic dermatitis. Clin. Rev. Allergy Immunol..

[153] Azfar R.S., Gelfand J.M. (2008). Psoriasis and metabolic disease: Epidemiology and pathophysiology. Curr. Opin. Rheumatol..

[154] Boehncke W.H. (2018). Systemic inflammation and cardiovascular comorbidity in psoriasis patients: Causes and consequences. Front. Immunol..

[155] Boehncke W.H., Schön M.P. (2015). Psoriasis. Lancet.

[156] Lande R., Gregorio J., Facchinetti V., Chatterjee B., Wang Y.H., Homey B., Cao W., Wang Y.H., Su B., Nestle F.O. (2007). Plasmacytoid dendritic cells sense self-DNA coupled with antimicrobial peptide. Nature.

[157] Zaba L.C., Fuentes-Duculan J., Eungdamrong N.J., Abello M.V., Novitskaya I., Pierson K.C., Gonzalez J., Krueger J.G., Lowes M.A. (2009). Psoriasis is characterized by accumulation of immunostimulatory and Th1/Th17 cell-polarizing myeloid dendritic cells. J. Investig. Dermatol..

[158] Lowes M.A., Russell C.B., Martin D.A., Towne J.E., Krueger J.G. (2013). The IL-23/T17 pathogenic axis in psoriasis is amplified by keratinocyte responses. Trends Immunol..

[159] Ferretti G., Alleva R., Taus M., Simonetti O., Cinti B., Offidani A.M., Bossi G., Curatola G. (1994). Abnormalities of plasma lipoprotein composition and fluidity in psoriasis. Acta Derm. Venereol..

[160] Ferretti G., Simonetti O., Offidani A.M., Messini L., Cinti B., Marshiseppe I., Bossi G., Curatola G. (1993). Changes of plasma lipids and erythrocyte membrane fluidity in psoriatic children. Pediatr. Res..

[161] Tam L.-S., Tomlinson B., Chu T.T.-W., Li M., Leung Y.-Y., Kwok L.-W., Li T.K., Yu T., Zhu Y.-E., Wong K.-C. (2008). Cardiovascular risk profile of patients with psoriatic arthritis compared to controls—the role of inflammation. Rheumatology.

[162] Mallbris L., Granath F., Hamsten A., Ståhle M. (2006). Psoriasis is associated with lipid abnormalities at the onset of skin disease. J. Am. Acad. Dermatol..

[163] Borska L., Kremlacek J., Andrys C., Krejsek J., Hamakova K., Borsky P., Palicka V., Rehacek V., Malkova A., Fiala Z. (2017). Systemic inflammation, oxidative damage to nucleic acids, and metabolic syndrome in the pathogenesis of psoriasis. Int. J. Mol. Sci..

[164] Nakhwa Y.C., Rashmi R., Basavaraj K.H. (2014). Dyslipidemia in Psoriasis: A Case Controlled Study. Int. Sch. Res. Not..

[165] Miao C., Li J., Li Y., Zhang X. (2019). Obesity and dyslipidemia in patients with psoriasis: A case-control study. Medicine (Baltimore).

[166] Ferdinando L.B., Fukumoto P.K., Sanches S., Fabricio L.H.Z., Skare T.L. (2018). Metabolic syndrome and psoriasis: A study in 97 patients. Rev. Assoc. Med. Bras..

[167] Pietrzak A., Chabros P., Grywalska E., Kiciński P., Franciszkiewicz-Pietrzak K., Krasowska D., Kandzierski G. (2019). Serum lipid metabolism in psoriasis and psoriatic arthritis—An update. Arch. Med. Sci..

[168] Pietrzak A., Kadzielewski J., Janowski K., Roliński J., Krasowska D., Chodorowska G., Paszkowski T., Kapeć E., Jastrzbska I., Tabarkiewicz J. (2009). Lipoprotein (a) in patients with psoriasis: Associations with lipid profiles and disease severity. Int. J. Dermatol..

[169] Pietrzak A., Grywalska E., Walankiewicz M., Lotti T., Roliński J., Myśliński W., Chabros P., Piekarska-Myślińska D., Reich K. (2017). Psoriasis and metabolic syndrome in children: Current data. Clin. Exp. Dermatol..

[170] Sarvtin M.T., Hedayati M.T., Tahereh Shokohi Z.H. (2014). Serum Lipids and Lipoproteins in Patients With Psoriasis. Arch. Iran Med..

[171] Khan S., Agrawal S., Baral N., Lamsal M. (2018). Evaluation of ADA activity as a potential marker of disease severity in psoriasis patients. Psoriasis Targets Ther..

[172] Sabry H.H., Sabry J.H., Daifalla A.E.H., Akl E.M., Hamed A.M., Torky A.A.A. (2018). Serum markers for asymptomatic atherosclerosis in Egyptian psoriatic patients: Study controlled by doppler estimation of carotid intima-media thickness. Vasc. Health Risk Manag..

[173] Cao L.Y., Soler D.C., Debanne S.M., Grozdev I., Rodriguez M.E., Feig R.L., Carman T.L., Gilkeson R.C., Orringer C.E., Kern E.F. (2014). Psoriasis and Cardiovascular Risk Factors: Increased Serum Myeloperoxidase and Corresponding Immunocellular Overexpression by Cd11b(+) CD68(+) Macrophages in Skin Lesions. Am. J. Transl. Res..

[174] Komorowska O., Bohdan M., Szczerkowska-Dobosz A., Rawicz-Zegrzda D., Dudziak M., Zdrojewski T., Gruchala M., Dorota Purzycka-Bohdan R.N. (2016). Assessment of Cardiovascular Risk Factors in Patients With Psoriasis. Acta Dermatovenerol. Croat..

[175] El Asmi M.A., Zidi W., Mebazaa A., Zayani Y., Ayadi I., Feki M., Osman A.B., Kaabachi N. (2014). Serum lipid level in tunisian patients with psoriasis. Clin. Lab..

[176] Pang X., Lin K., Liu W., Zhang S.Z. (2015). Characterization of the Abnormal Lipid Profile in Chinese Patients With Psoriasis. Int. J. Clin. Exp. Pathol..

[177] Akkara Veetil B.M., Matteson E.L., Maradit-Kremers H., McEvoy M.T., Crowson C.S. (2012). Trends in lipid profiles in patients with psoriasis: A population-based analysis. BMC Dermatol..

[178] Coimbra S., Oliveira H., Reis F., Belo L., Rocha S., Quintanilha A., Figueiredo A., Teixeira F., Castro E., Rocha-Pereira P. (2010). Psoriasis therapy and cardiovascular risk factors: A 12-week follow-up study. Am. J. Clin. Dermatol..

[179] Yuksel E.P., Yuksel S., Yenercag M., Soylu K., Aydin F., Senturk N., Yucel H., Canturk T., Turanli A.Y. (2014). Impaired heart rate recovery indices in psoriasis patients. Med. Sci. Monit..

[180] Mebazaa A., El Asmi M., Zidi W., Zayani Y., Cheikh Rouhou R., El Ounifi S., Kanoun F., Mokni M., Osman A.B., Feki M. (2011). Metabolic syndrome in Tunisian psoriatic patients: Prevalence and determinants. J. Eur. Acad. Dermatol. Venereol..

[181] Sirin M.C., Korkmaz S., Erturan I., Filiz B., Aridogan B.C., Cetin E.S., Yildirim M. (2020). Evaluation of monocyte to HDL cholesterol ratio and other inflammatory markers in patients with psoriasis. An. Bras. Dermatol..

[182] Bajaj S., Mandal S., Singh K.G., Prajapati R. (2020). Metabolic Diseases and Associated Complications in Patients with Psoriasis. J. Assoc. Physicians India.

[183] Rocha-Pereira P., Santos-Silva A., Rebelo I., Figueiredo A., Quintanilha A., Teixeira F. (2001). Dislipidemia and oxidative stress in mild and in severe psoriasis as a risk for cardiovascular disease. Clin. Chim. Acta.

[184] Tekin N.S., Tekin I.O., Barut F., Sipahi E.Y. (2007). Accumulation of oxidized low-density lipoprotein in psoriatic skin and changes of plasma lipid levels in psoriatic patients. Mediat. Inflamm..

[185] He L., Qin S., Dang L., Song G., Yao S., Yang N., Li Y. (2014). Psoriasis decreases the anti-oxidation and anti-inflammation properties of high-density lipoprotein. Biochim. Biophys. Acta Mol. Cell Biol. Lipids.

[186] Usta M., Turan E., Aral H., Inal B.B., Gurel M.S., Guvenen G. (2011). Serum paraoxonase-1 activities and oxidative status in patients with plaque-type psoriasis with/without metabolic syndrome. J. Clin. Lab. Anal..

[187] Reynoso-von Drateln C., Martínez-Abundis E., Balcázar-Muñoz B.R., Bustos-Saldaña R., González-Ortiz M. (2003). Lipid profile, insulin secretion, and insulin sensitivity in psoriasis. J. Am. Acad. Dermatol..

[188] Thungaturthi S., Vadakedath S., Pavuluri P., Rani J., Gundu R., Bheem J., Kandi V. (2019). Atherogenesis in Psoriasis: Evaluation of the Serum Activities of Non-high-density Lipoprotein Cholesterol and Other Lipids Among Newly Diagnosed Psoriasis Patients. Cureus.

[189] Uyanik B.S., Ari Z., Onur E., Gündüz K., Tanülkü S., Durkan K. (2002). Serum lipids and apolipoproteins in patients with psoriasis. Clin. Chem. Lab. Med..

[190] Shreberk-Hassidim R., Galili E., Hassidim A., Ramot Y., Merdler I., Baum S., Zlotogorski A., Barzilai A., Astman N. (2019). Epidemiology and Comorbidities of Psoriasis among Israeli Adolescents: A Large Cross-Sectional Study. Dermatology.

[191] Asha K., Singal A., Sharma S.B., Arora V.K., Aggarwal A. (2017). Dyslipidaemia & oxidative stress in patients of psoriasis: Emerging cardiovascular risk factors. Indian J. Med. Res..

[192] Madanagobalane S., Anandan S. (2012). Prevalence of metabolic syndrome in south Indian patients with psoriasis vulgaris and the relation between disease severity and metabolic syndrome: A hospital-based case-control study. Indian J. Dermatol..

[193] Uczniak S., Gerlicz Z.A., Kozłowska M., Kaszuba A. (2016). Presence of selected metabolic syndrome components in patients with psoriasis vulgaris. Postep. Dermatol. Alergol..

[194] Akcali C., Buyukcelik B., Kirtak N., Inaloz S. (2014). Clinical and laboratory parameters associated with metabolic syndrome in Turkish patients with psoriasis. J. Int. Med. Res..

[195] Zindancı I., Albayrak O., Kavala M., Kocaturk E., Can B., Sudogan S., Koç M. (2012). Prevalence of Metabolic Syndrome in Patients with Psoriasis. Sci. World J..

[196] Love T.J., Qureshi A.A., Karlson E.W., Gelfand J.M., Choi H.K. (2011). Prevalence of the metabolic syndrome in psoriasis: Results from the national health and nutrition examination survey, 2003–2006. Arch. Dermatol..

[197] Miller I.M., Skaaby T., Ellervik C., Jemec G.B.E. (2013). Quantifying cardiovascular disease risk factors in patients with psoriasis: A meta-analysis. Br. J. Dermatol..

[198] Piskin S., Gurkok F., Ekuklu G., Senol M. (2003). Serum lipid levels in psoriasis. Yonsei Med. J..

[199] Seçkin D., Tokgözoğlu L., Akkaya S. (1994). Are lipoprotein profile and lipoprotein (a) levels altered in men with psoriasis?. J. Am. Acad. Dermatol..

[200] Akhyani M., Ehsani A.H., Robati R.M., Robati A.M. (2007). The lipid profile in psoriasis: A controlled study. J. Eur. Acad. Dermatol. Venereol..

[201] Seishima M., Seishima M., Mori S., Noma A. (1994). Serum lipid and apolipoprotein levels in patients with psoriasis. Br. J. Dermatol..

[202] Farshchian M., Zamanian A., Farshchian M., Monsef A.-R., Mahjub H. (2007). Serum lipid level in Iranian patients with psoriasis. J. Eur. Acad. Dermatol. Venereol..

[203] Ferretti G., Bacchetti T., Campanati A., Simonetti O., Liberati G., Offidani A. (2012). Correlation between lipoprotein(a) and lipid peroxidation in psoriasis: Role of the enzyme paraoxonase-1. Br. J. Dermatol..

[204] Toker A., Kadi M., Yildirim A.K., Aksoy H., Akçay F. (2009). Serum lipid profile paraoxonase and arylesterase activities in psoriasis. Cell Biochem. Funct..

[205] Sorokin A.V., Kotani K., Elnabawi Y.A., Dey A.K., Sajja A.P., Yamada S., Ueda M., Harrington C.L., Baumer Y., Rodante J.A. (2018). Association between oxidation-modified lipoproteins and coronary plaque in psoriasis an observational cohort study. Circ. Res..

[206] Golden J.B., McCormick T.S., Ward N.L. (2013). IL-17 in psoriasis: Implications for therapy and cardiovascular co-morbidities. Cytokine.

[207] Wolk R., Armstrong E.J., Hansen P.R., Thiers B., Lan S., Tallman A.M., Kaur M., Tatulych S. (2017). Effect of tofacitinib on lipid levels and lipid-related parameters in patients with moderate to severe psoriasis. J. Clin. Lipidol..

[208] Wu J.J., Strober B.E., Hansen P.R., Ahlehoff O., Egeberg A., Qureshi A.A., Robertson D., Valdez H., Tan H., Wolk R. (2016). Effects of tofacitinib on cardiovascular risk factors and cardiovascular outcomes based on phase III and long-term extension data in patients with plaque psoriasis. J. Am. Acad. Dermatol..

[209] Papp K.A., Menter A., Strober B., Langley R.G., Buonanno M., Wolk R., Gupta P., Krishnaswami S., Tan H., Harness J.A. (2012). Efficacy and safety of tofacitinib, an oral Janus kinase inhibitor, in the treatment of psoriasis: A Phase 2b randomized placebo-controlled dose-ranging study. Br. J. Dermatol..

[210] Singh S., Bhansali A. (2017). Randomized placebo control study of metformin in psoriasis patients with metabolic syndrome (systemic treatment cohort). Indian J. Endocrinol. Metab..

[211] Zangrilli A., Bavetta M., Scaramella M., Bianchi L. (2018). Long-term treatment of psoriatic patients with adalimumab reduces disease severity and maintains a favorable lipid pattern and a low Atherogenic Index. G. Ital. Dermatol. Venereol..

[212] Puig L., Strohal R., Fuiman J., Pedersen R., Szumski A., Koenig A.S., Robertson D., Drexel H. (2014). Cardiometabolic biomarkers in chronic plaque psoriasis before and after etanercept treatment. J. Dermatolog. Treat..

[213] Dey A.K., Joshi A.A., Chaturvedi A., Lerman J.B., Aberra T.M., Rodante J.A., Teague H.L., Harrington C.L., Rivers J.P., Chung J.H. (2017). Association between skin and aortic vascular inflammation in patients with psoriasis: A case-cohort study using positron emission tomography/computed tomography. JAMA Cardiol..

[214] Kilic S., Emre S., Metin A., Isikoglu S., Erel O. (2013). Effect of the systemic use of methotrexate on the oxidative stress and paraoxonase enzyme in psoriasis patients. Arch. Dermatol. Res..

[215] Corbetta S., Angioni R., Cattaneo A., Becke-Peccoz P., Spada A. (2006). Effects of retinoid therapy on insulin sensitivity, lipid profile and circulating adipocytokines. Eur. J. Endocrinol..

[216] Bacchetti T., Campanati A., Ferretti G., Simonetti O., Liberati G., Offidani A.M. (2013). Oxidative stress and psoriasis: The effect of antitumour necrosis factor-α inhibitor treatment. Br. J. Dermatol..

[217] Egeberg A., Wu J.J., Korman N., Solomon J.A., Goldblum O., Zhao F., Mallbris L. (2018). Ixekizumab treatment shows a neutral impact on cardiovascular parameters in patients with moderate-to-severe plaque psoriasis: Results from UNCOVER-1, UNCOVER-2, and UNCOVER-3. J. Am. Acad. Dermatol..

[218] Gelfand J.M., Shin D.B., Duffin K.C., Armstrong A.W., Blauvelt A., Tyring S.K., Menter A., Gottlieb S., Lockshin B.N., Simpson E.L. (2020). A Randomized Placebo-Controlled Trial of Secukinumab on Aortic Vascular Inflammation in Moderate-to-Severe Plaque Psoriasis (VIP-S). J. Investig. Dermatol..

[219] Ahlehoff O., Hansen P.R., Gislason G.H., Frydland M., Bryld L.E., Elming H., Jemec G.B.E. (2016). Myocardial function and effects of biologic therapy in patients with severe psoriasis: A prospective echocardiographic study. J. Eur. Acad. Dermatol. Venereol..

[220] Mehta N.N., Shin D.B., Joshi A.A., Dey A.K., Armstrong A.W., Duffin K.C., Fuxench Z.C., Harrington C.L., Hubbard R.A., Kalb R.E. (2018). Effect of 2 psoriasis treatments on vascular inflammation and novel inflammatory cardiovascular biomarkers: A randomized placebo-controlled trial. Circ. Cardiovasc. Imaging.

[221] Staniak H.L., Bittencourt M.S., de Souza Santos I., Sharovsky R., Sabbag C., Goulart A.C., Lotufo P.A., Benseñor I.M. (2014). Association between psoriasis and coronary calcium score. Atherosclerosis.

[222] Asefi M., Vaisi-Raygani A., Bahrehmand F., Kiani A., Rahimi Z., Nomani H., Ebrahimi A., Tavilani H., Pourmotabbed T. (2012). Paraoxonase 1 (PON1) 55 polymorphism, lipid profiles and psoriasis. Br. J. Dermatol..

[223] Houshang N., Reza K., Sadeghi M., Ali E., Mansour R., Vaisi-Raygani A. (2014). Antioxidant status in patients with psoriasis. Cell Biochem. Funct..

[224] Bacchetti T., Simonetti O., Ricotti F., Offidani A., Ferretti G. (2020). Plasma oxidation status and antioxidant capacity in psoriatic children. Arch. Dermatol. Res..

[225] Husni M.E., Wilson Tang W.H., Lucke M., Chandrasekharan U.M., Brennan D.M., Hazen S.L. (2018). Correlation of High-Density Lipoprotein–Associated Paraoxonase 1 Activity With Systemic Inflammation, Disease Activity, and Cardiovascular Risk Factors in Psoriatic Disease. Arthritis Rheumatol..

[226] Zuberbier T., Asero R., Bindslev-Jensen C., Walter Canonica G., Church M.K., Giménez-Arnau A.M., Grattan C.E.H., Kapp A., Maurer M., Merk H.F. (2009). EAACI/GALEN/EDF/WAO guideline: Management of urticaria. Allergy: European Journal of Allergy and Clinical Immunology.

[227] Kanani A., Betschel S.D., Warrington R. (2018). Urticaria and angioedema. Allergy Asthma Clin. Immunol..

[228] Zuberbier T., Balke M., Worm M., Edenharter G., Maurer M. (2010). Epidemiology of urticaria: A representative cross-sectional population survey. Clin. Exp. Dermatol..

[229] Maurer M., Ortonne J.P., Zuberbier T. (2009). Chronic urticaria: An internet survey of health behaviours, symptom patterns and treatment needs in European adult patients. Br. J. Dermatol..

[230] Amar S.M., Dreskin S.C. (2008). Urticaria. Prim. Care Clin. Off. Pract..

[231] Antia C., Baquerizo K., Korman A., Bernstein J.A., Alikhan A. (2018). Urticaria: A comprehensive review: Epidemiology, diagnosis, and work-up. J. Am. Acad. Dermatol..

[232] Amin M.M., Rushdy M. (2018). Hyperlipidemia in association with pro-inflammatory cytokines among chronic spontaneous urticaria: Case-control study. Eur. Ann. Allergy Clin. Immunol..

[233] Yaldiz M., Asil K. (2020). Evaluation of carotid intima media thickness and hematologic inflammatory markers in patients with chronic spontaneous urticaria. Adv. Dermatol. Allergol..

[234] Kaplan A.P., Greaves M.W. (2005). Angioedema. J. Am. Acad. Dermatol..

[235] Sloane D.E., Lee C.W., Sheffer A.L. (2007). Hereditary angioedema: Safety of long-term stanozolol therapy. J. Allergy Clin. Immunol..

[236] Széplaki G., Varga L., Valentin S., Kleiber M., Karádi I., Romics L., Füst G., Farkas H. (2005). Adverse effects of danazol prophylaxis on the lipid profiles of patients with hereditary angioedema. J. Allergy Clin. Immunol..

[237] Szegedi R., Széplaki G., Varga L., Prohászka Z., Széplaki Z., Karádi I., Füst G., Farkas H. (2008). Long-term danazol prophylaxis does not lead to increased carotid intima-media thickness in hereditary angioedema patients. Atherosclerosis.

[238] Nebenführer Z., Szabó E., Kajdácsi E., Kőhalmi K.V., Karádi I., Zsáry A., Farkas H., Cervenak L. (2019). Flow-mediated vasodilation assay indicates no endothelial dysfunction in hereditary angioedema patients with C1-inhibitor deficiency. Ann. Allergy Asthma Immunol..

[239] Wurfel M.M., Kunitake S.T., Lichenstein H., Kane J.P., Wright S.D. (1994). Lipopolysaccharide (LPS)-binding protein is carried on lipoproteins and acts as a cofactor in the neutralization of LPS. J. Exp. Med..

[240] Levels J.H.M., Abraham P.R., Van Barreveld E.P., Meijers J.C.M., Van Deventer S.J.H. (2003). Distribution and kinetics of lipoprotein-bound lipoteichoic acid. Infect. Immun..

[241] Levels J.H.M., Abraham P.R., Van den Ende A., Van Deventer S.J.H. (2001). Distribution and kinetics of lipoprotein-bound endotoxin. Infect. Immun..

[242] Curcic S., Holzer M., Frei R., Pasterk L., Schicho R., Heinemann A., Marsche G. (2015). Neutrophil effector responses are suppressed by secretory phospholipase A2 modified HDL. Biochim. Biophys. Acta Mol. Cell Biol. Lipids.

